# Ubiquitin-Regulated Nuclear-Cytoplasmic Trafficking of the Nipah Virus Matrix Protein Is Important for Viral Budding

**DOI:** 10.1371/journal.ppat.1001186

**Published:** 2010-11-11

**Authors:** Yao E. Wang, Arnold Park, Michael Lake, Mickey Pentecost, Betsabe Torres, Tatyana E. Yun, Mike C. Wolf, Michael R. Holbrook, Alexander N. Freiberg, Benhur Lee

**Affiliations:** 1 Department of Microbiology, Immunology, and Molecular Genetics, UCLA, Los Angeles, California, United States of America; 2 Department of Pathology, University of Texas Medical Branch, Galveston, Texas, United States of America; 3 Integrated Research Facility, National Institutes of Health, National Institute of Allergy and Infectious Diseases, Frederick, Maryland, United States of America; 4 Department of Pathology and Laboratory Medicine, UCLA, Los Angeles, California, United States of America; 5 UCLA AIDS Institute, UCLA, Los Angeles, California, United States of America; Mount Sinai School of Medicine, United States of America

## Abstract

Paramyxoviruses are known to replicate in the cytoplasm and bud from the plasma membrane. Matrix is the major structural protein in paramyxoviruses that mediates viral assembly and budding. Curiously, the matrix proteins of a few paramyxoviruses have been found in the nucleus, although the biological function associated with this nuclear localization remains obscure. We report here that the nuclear-cytoplasmic trafficking of the Nipah virus matrix (NiV-M) protein and associated post-translational modification play a critical role in matrix-mediated virus budding. Nipah virus (NiV) is a highly pathogenic emerging paramyxovirus that causes fatal encephalitis in humans, and is classified as a Biosafety Level 4 (BSL4) pathogen. During live NiV infection, NiV-M was first detected in the nucleus at early stages of infection before subsequent localization to the cytoplasm and the plasma membrane. Mutations in the putative bipartite nuclear localization signal (NLS) and the leucine-rich nuclear export signal (NES) found in NiV-M impaired its nuclear-cytoplasmic trafficking and also abolished NiV-M budding. A highly conserved lysine residue in the NLS served dual functions: its positive charge was important for mediating nuclear import, and it was also a potential site for monoubiquitination which regulates nuclear export of the protein. Concordantly, overexpression of ubiquitin enhanced NiV-M budding whereas depletion of free ubiquitin in the cell (via proteasome inhibitors) resulted in nuclear retention of NiV-M and blocked viral budding. Live Nipah virus budding was exquisitely sensitive to proteasome inhibitors: bortezomib, an FDA-approved proteasome inhibitor for treating multiple myeloma, reduced viral titers with an IC_50_ of 2.7 nM, which is 100-fold less than the peak plasma concentration that can be achieved in humans. This opens up the possibility of using an “off-the-shelf” therapeutic against acute NiV infection.

## Author Summary

Nipah virus (NiV) is a lethal, newly emerging virus that causes fatal inflammation of the brain and has a high death rate in infected humans. NiV and the closely related Hendra virus (HeV) can also infect agriculturally important livestock such as pigs and horses. The lack of effective vaccines and treatments, and the ongoing threat they pose to both agriculture and public health, have led to the classification of NiV and HeV as Biosafety Level 4 (BSL4) pathogens. Paramyxoviruses such as NiV are known to replicate in the cytoplasm and bud from the plasma membrane. Viral assembly and budding is mediated by the matrix structural protein. However, we found, quite unexpectedly, that the matrix protein of NiV needs to transit through the nucleus before gaining the functional ability to localize and bud from the plasma membrane. Although NiV-M has putative nuclear import and export signals, we also found that ubiquitination of a conserved lysine residue in NiV-M is critical for nuclear export, subsequent membrane localization and viral budding. Proteasome inhibitors, which deplete cellular pools of free ubiquitin, potently reduce viral titers during live NiV infection, opening up new possibilities for therapeutics against acute NiV infection.

## Introduction

Nipah virus (NiV) is a highly pathogenic paramyxovirus that has recently emerged from fruit bats to cause fatal diseases in humans [1], [2], [3]. It was first identified as the etiologic agent responsible for an outbreak of severe encephalitis in Malaysia and Singapore that began in 1998 and continued into 1999 with a case-fatality rate of 40% [3]. In the initial cases of NiV infection, the virus is thought to have transmitted from pigs to humans, although it is able to infect a broad spectrum of animal hosts under natural and experimental conditions [1], [4]. Later outbreaks of NiV encephalitis in Bangladesh were associated with an increased mortality rate (up to 75%), and there has been evidence for direct human-to-human transmission [5]. The high virulence of the viruses and the absence of effective therapeutic modalities and vaccines have led to the classification of NiV and the closely-related Hendra virus (HeV) as Biosafety Level 4 (BSL4) pathogens [1]. Indeed, recent outbreaks of Hendra virus in Queensland, Australia (Aug-Sep 2009) have killed 3 horses and one veterinarian, and led to the quarantine of affected horse farms and potentially infected individuals [6] **.** Thus, NiV and HeV infections pose an ongoing threat to both agriculture and public health.

NiV and HeV comprise a new genus Henipavirus within the family *Paramyxoviridae.* This is a family of viruses with negative-stranded RNA genomes and lipid envelopes derived from the host cell membrane. The genome contains six principle genes: nucleocapsid (N), phosphoprotein (P), polymerase (L), matrix (M), fusion (F) and attachment (HN, H or G) proteins [7]. Paramyxoviruses are known to replicate in the cytoplasm, and progeny virions are released from the plasma membrane of the host cell. Viral assembly and budding are orchestrated by the matrix protein (M), a major structural protein underlying the viral envelope [7], [8], [9]. Previous studies have shown that when expressed alone in the cell, NiV-M in itself carries sufficient information for the spontaneous formation and release of viral-like particles (VLPs) in the absence of other viral components [10], [11], [12]. However, despite the identification of the YMYL motif in NiV-M as a potential late-domain [10] and the YPLGVG motif as another requirement for budding [12], the intracellular trafficking and budding pathways of NiV-M remain poorly defined. In our attempt to characterize the trafficking pathway of NiV-M, we found, quite unexpectedly, that it translocates to the nucleus at early stages of infection before localizing to the plasma membrane, suggesting a previously unappreciated role for the nuclear-cytoplasmic trafficking of the Nipah matrix protein in the viral life cycle.

Though paramyxoviruses replicate in the cytoplasm, nuclear localization of viral accessory proteins has been described before. For example, the W protein of NiV inhibits host interferon response by sequestering STAT1 in the nucleus [13], [14], [15], and a fraction of the V protein of human parainfluenza virus type 2 can be found in the nucleus [16]. The nuclear localization of viral structural proteins, however, is less expected. Within *Paramyxoviridae*, the matrix protein has been reported to localize to the nuclear compartment in three cases so far: Sendai virus (SeV) [17], Newcastle disease virus (NDV) [18], [19], [20] and human respiratory syncytial virus (HRSV) [21], [22], [23]. In SeV and NDV, although the nuclear localization of M was clearly described, the biological function of this nuclear localization remains undefined. Faaberg et al examined more than 10 strains of NDV and found that the degree of M nuclear localization appears unrelated to virulence *per se* [24]. In the case of HRSV, Ghildyal et al showed that nuclear extract from HRSV-infected cells supports in vitro transcription less efficiently compared to mock-infected cells, but it has yet to be demonstrated that this inhibition is directly attributable to M [23]. A recent study on HRSV showed that Crm1-dependent nuclear export of the matrix protein is important for viral assembly and budding, suggesting that nuclear trafficking of M is somehow involved in effectuating proper viral budding [21]. However, it remains unclear why M budding needs a nuclear transit phase or whether M's nuclear localization has additional biological functions.

For proteins larger than 40 kD, efficient transport across the nuclear membrane is mediated by specific import and export signals [25], [26]. There are two types of nuclear localization signals (NLSs) that have been well characterized. A monopartite NLS consists of one single cluster of positively charged amino acid residues such as lysine (K) or arginine (R), whereas a bipartite NLS contains two stretches of K/R residues conforming to the consensus (K/R)(K/R)-X_10–12_-(K/R)(K/R) (where X stands for any amino acid residue) [27], [28], [29]. Nuclear export signals (NESs) are less well-defined although leucine/isoleucine-rich stretches have been identified as NESs. The most well-defined nuclear export pathway involves the chromosomal region maintenance protein 1 (CRM-1), which recognizes these leucine/isoleucine-rich NESs [30], [31]. Additionally, post-translational modifications such as ubiquitination and SUMOylation have also been shown to regulate the nuclear-cytoplasmic trafficking of cellular proteins including p53, NF-kB, PTEN, and NEMO [32], [33], [34], [35], [36], although their involvement in viral protein trafficking is less known. Nevertheless, many viruses have evolved to co-opt the cellular ubiquitin/proteasome system as a means of manipulating the host cell cycle, evading the immune system as well as egressing from the infected cell [37], [38].

Here, we find in one viral structural protein, the use and convergence of three well known cellular pathways for nuclear-cytoplasmic trafficking. We report that proper nuclear-cytoplasmic trafficking of NiV-M is essential for viral budding, and that NiV-M's nuclear-cytoplasmic trafficking is regulated by a putative bipartite NLS, a leucine-rich NES, as well as potential ubiquitination on a conserved lysine residue located in the bipartite NLS itself. Not only does this lysine play key roles in both nuclear import and export, it is also indispensable for the plasma membrane targeting of NiV-M and its subsequent incorporation into virions. Live Nipah virus budding is exquisitely sensitive to ubiquitin depletion, which leads to the nuclear retention of NiV-M. Our results suggest the clinical use of FDA-approved proteasome inhibitors such as bortezomib (Velcade) as a potential “off-the-shelf” therapeutic against acute NiV infection.

## Results

### Nipah virus matrix protein transits through the nuclear compartment before it localizes to the plasma membrane

In order to examine the subcellular localization of Nipah virus matrix protein (NiV-M) during the natural course of viral infection, NiV-infected cells were fixed at different time points post-infection and processed for analysis by confocal microscopy. The polyclonal anti-NiV-M antibody used in these experiments was raised by immunizing rabbits with a peptide corresponding to amino acids 29–49 of NiV-M. We verified that this affinity purified antibody was highly specific to NiV-M and had very low background staining in M non-expressing cells (Fig. S1).

**Figure 1:**
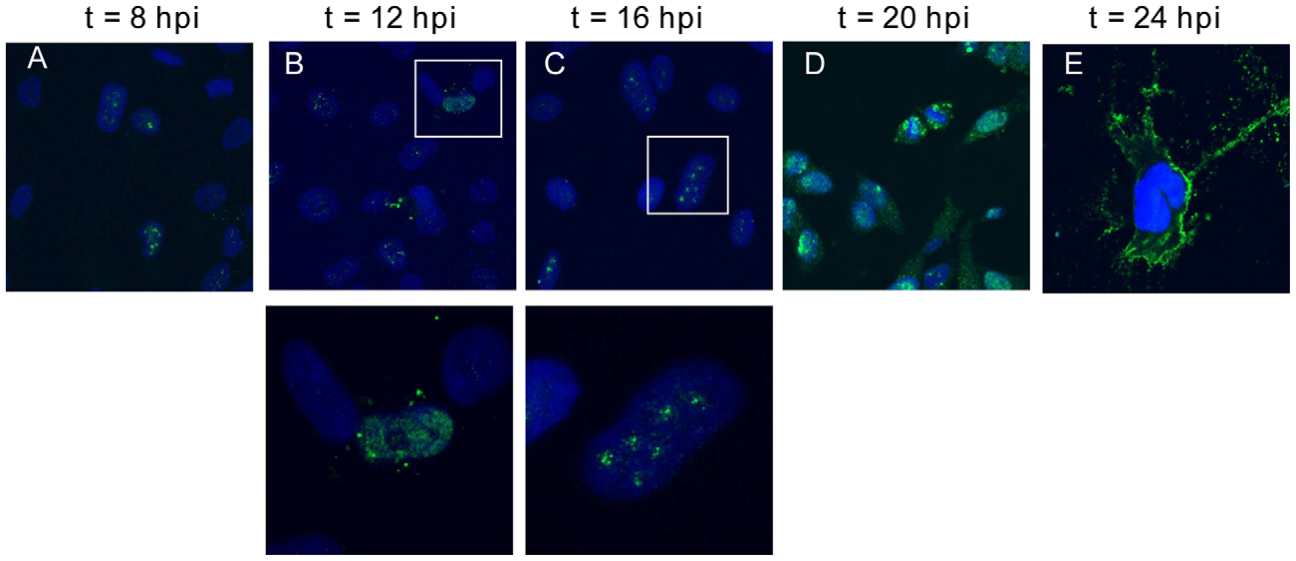
Nuclear-cytoplasmic trafficking of Nipah virus matrix protein (NiV-M) during live viral infection. HeLa cells plated on poly-lysine-coated glass coverslips were incubated with Nipah virus Malaysia strain for 1 hr at 37°C and then fresh growth medium for up to 24 hrs. At (**A**) 8, (**B**) 12, (**C**) 16, (**D**) 20, and (**E**) 24 hpi, cells were fixed with 10% formalin, stained with rabbit anti-M polyclonal antibody and imaged on a confocal fluorescent microscope (63× magnification). DAPI was used for visualization of the nuclei. Insets in (B) and (C) indicate nuclear localization of M in infected cells. Experiments were performed under BSL4 conditions.

At early time points (between 8 and 16 hrs) post-infection, many cells had M protein primarily concentrated in the nuclei and fluorescence followed a discrete punctuate staining pattern (Figs. 1A to C). At later time points (20 to 24 hrs), NiV-M protein was distributed diffusely in both the cytoplasm and nucleus of infected cells (Figs. 1D and E). At the latest time-point examined (24 hrs), when syncytia have begun to form, NiV-M was more clearly localized to patches on the plasma membrane and filamentous membrane extensions.

To facilitate further biochemical characterizations and mutagenesis studies of NiV-M, we generated an N-terminally triple FLAG-tagged NiV-M expression construct (3XFLAG-M). This tagged protein, when expressed alone in HeLa cells, exhibited similar localization patterns as those seen during the natural course of viral infection. It concentrated in the nuclear compartment before the cytoplasmic staining became prominent (Fig. S2). A GFP-M fusion protein also behaved in a similar manner (data not shown).

### NiV-M possesses a putative bipartite nuclear localization signal (NLS) and a leucine-rich nuclear export signal (NES)

The calculated molecular weight of NiV-M is 39 kD, which is around the upper limit for free diffusion across the nuclear envelope. Efficient nuclear import and export of proteins >20–40 kD usually require nuclear localization signals (NLSs) and nuclear export signals (NESs), respectively [26]. Sequence analysis of NiV-M revealed the presence of one cluster of positively charged amino acids analogous to known monopartite NLSs as well as a potential bipartite NLS consisting of two short stretches of lysines/arginines separated by ten other amino acid residues (Table 1). There are also two leucine/isoleucine-rich stretches in NiV-M that conform to the consensus for NESs (Table 2).

**Table 1:** Alignment of Nipah matrix sequence with known NLSs.

Monopartite NLS consensus	short stretch of K/R
SV40 T Antigen	P **KKKRK** V
Histone H2B	G**KKR**S**K**V
NiV matrix	^82^**KRKK**I**R**^87^

Bipartite NLS consensus	(K/R)(K/R)-X_10-12_-(K/R)(K/R)
Nucleoplasmin	**KR**PAATKKAGQA**KKKK**LDK
Human p53	**KR**ALPNNTSSSPQP**KKK**P
NiV-M	**^244^RR**AGKYYSVDYC **RRK^258^**

(Note: NLS =  Nuclear Localization Signal; K = lysine; R = arginine; X = any amino acid residue. The positively-charged amino acid residues in each NLS are in bold.)

**Table 2:** Alignment of Nipah matrix sequence with known NESs.

NES consensus	L-X_2-3_-L-X_2-3_-L-X-L
HIV Rev	**L**PP**L**ER**L**T**L**
Ad5 E1B	**L**YPE**L**RRI**L**T**I**
NiV-M (N-ter)	**^106^ LL**EE**L**CS**L**KV**^115^**
NiV-M (C-ter)	**^268^L**GS**I**GG**L**S**L^276^**

(Note: NES =  Nuclear Export Signal; L = Leucine; I = Isoleucine; X = any amino acid residue. The key L/I residues in each NES are in bold.)

**Figure 2:**
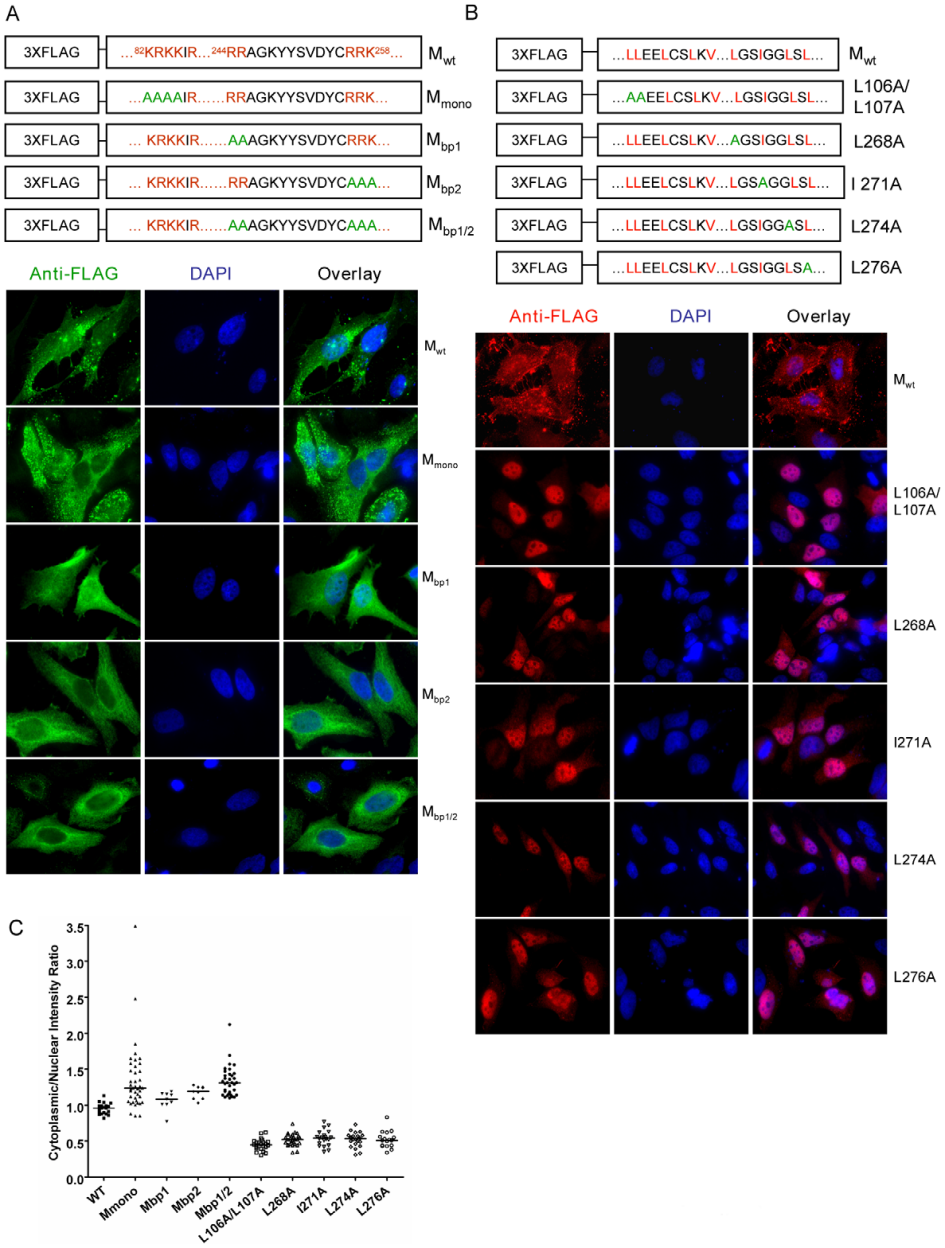
Mutagenesis studies of potential nuclear localization signals (NLSs) and nuclear export signals (NESs) in NiV-M. Positively charged amino acid residues in the predicted monopartite and bipartite NLSs (**A**) or key leucine/isoleucine residues in the potential NESs (**B**) were mutated to alanines using site-directed mutagenesis. HeLa cells expressing the indicated proteins were stained with an anti-FLAG monoclonal antibody as well as DAPI. Representative fields are shown in (A) and (B), and (**C**) shows the quantification of cytoplasmic/nuclear fluorescence intensity (C:N) ratios for ∼10–50 individual cells analyzed for each mutant as described in *Materials and Methods*. Compared to Mwt, statistically significant increases in C:N ratios were observed for M_bp1_ (p<0.01), M_bp2_ (p<0.0001) and M_bp1/2_ (p<0.0001) (unpaired t-test).

To test whether the NLSs are functional, we performed alanine substitution of key lysine/arginine residues. Subcellular localization of these mutants was examined by immunofluorescence microscopy (Fig. 2A), and quantification of the cytoplasmic/nuclear fluorescence intensity ratio was performed as described in *Materials and Methods*. The monopartite NLS mutant did not give a very obvious phenotype compared to the wild-type matrix protein and showed large cell-to-cell variations. This mutant was therefore excluded from further analysis. Mutating the first part of the bipartite signal (M_bp1_) led to a mild nuclear exclusion phenotype, whereas mutating the second part (M_bp2_) had a more apparent effect. When both parts of the bipartite NLS were mutated (M_bp1/2_), nuclear import was most obviously impaired (Fig. 2A). These visual differences were confirmed by the quantification of the cytoplasmic/nuclear fluorescence intensity (C∶N) ratios shown in Fig. 2C. Note that M_bp1_, M_bp2_, and M_bp1/2_ had statistically significant increases in C∶N ratios compared to M_wt_, indicating increased cytoplasmic retention relative to nuclear import. Interestingly, we also noticed that while M_wt_ localized to punctuate structures in the cytoplasm as well as patches on the plasma membrane, the bi-partite NLS mutants, especially M_bp2_ and M_bp1/2,_ exhibited more diffused localization patterns.

**Figure 3:**
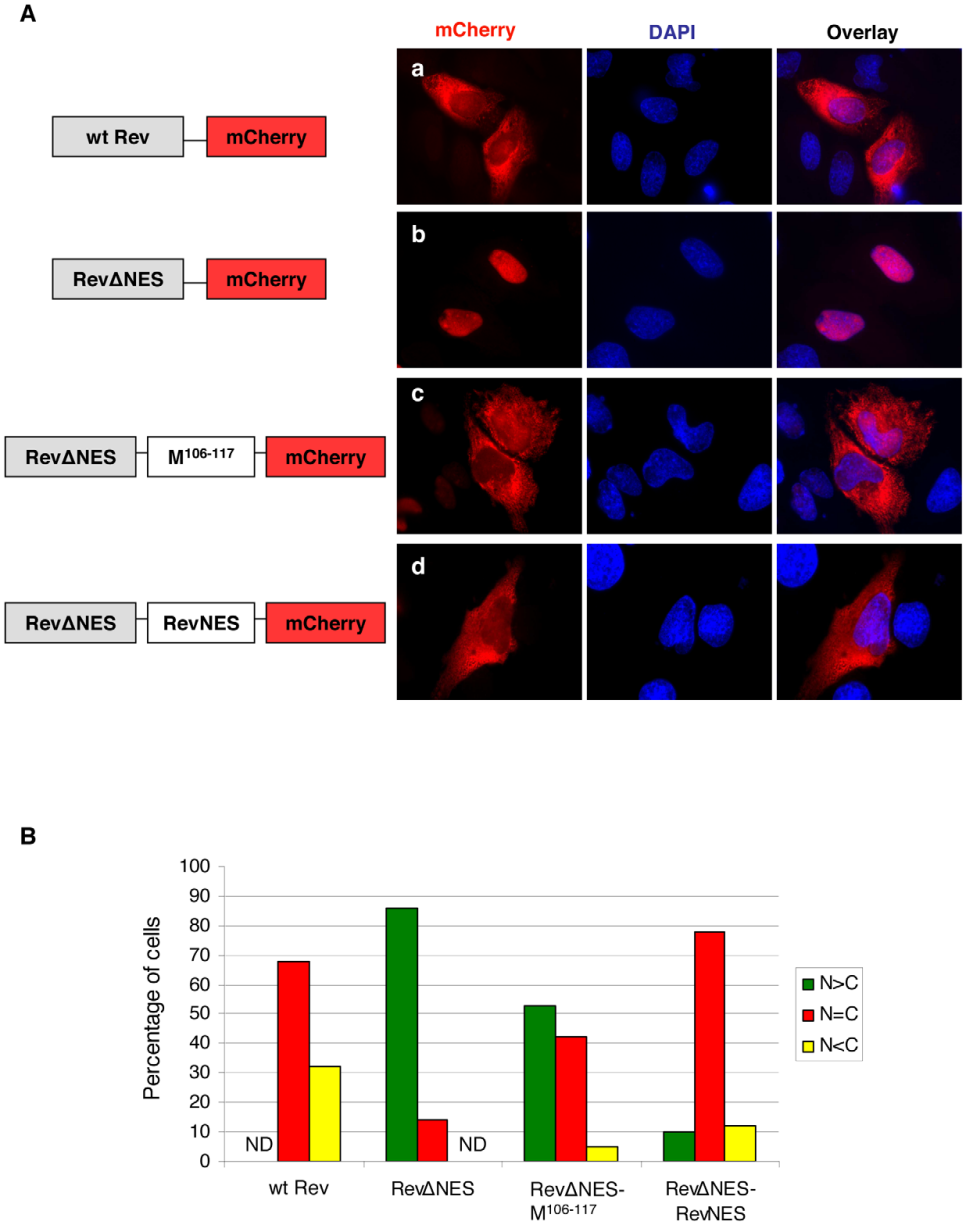
NiV-M NES partially restores nuclear export to an NES-defective HIV Rev. (**A**) HeLa cells were transiently transfected with plasmids encoding Rev-mCherry (panel a), RevΔNES-mCherry (panel b), RevΔNES-M^106–117^-mCherry (panel c), or RevΔNES-RevNES-mCherry (panel d). 24 hrs post transfection, cells were treated with 5 µg/ml actinomycin D for 4 hrs before fixation. Cells were stained with DAPI for visualization of the nuclei and imaged on a fluorescent microscope under 60× magnification. Representative images are shown in (A), and **(B)** shows the quantification of the percentage of cells with the fusion protein localized to only the nucleus (N>C), both the nucleus and the cytoplasm (N = C), or only the cytoplasm (N<C). For each mutant, at least 100 cells were counted. Both M^106–117^ and the endogenous NES from Rev were able to restore nuclear export to the RevΔNES-mCherry fusion protein.

Similarly, the key leucine/isoleucine residues in the potential NESs were mutated to alanines individually. All the NES mutants demonstrated nuclear retention phenotypes (Figs. 2B and C). However, previous studies have shown that deletion of the YMYL motif or the YPLGVG motif, originally thought to be late domain motifs, and neither of which conforms to a classical nuclear export sequence, also resulted in the nuclear retention of NiV-M [10], [12]. To test whether the two putative NESs in NiV-M are functional in the context of a heterologous protein, we adopted an experimental system similar to that developed by Henderson *et al* [39]. A fluorescent protein mCherry was fused to the C-terminus of the HIV Rev protein. This fusion protein localized to both the nucleus and the cytoplasm (Fig. 3A, panel a). When the endogenous NES in Rev was mutated (RevΔNES), the resulting fusion protein was restricted to the nuclear compartment (Fig. 3A, panel b), whereas the insertion of a short peptide corresponding to the first putative NES of NiV-M (amino acids 106–117) between RevΔNES and mCherry partially restored nuclear export (Fig. 3A, panel c). Insertion of a peptide corresponding to the second putative NES of NiV-M (amino acids 264–280) did not result in significant nuclear export of the fusion protein (data not shown). As a control, the endogenous Rev NES was inserted in the place of NiV-M NES, which led to significant nuclear export (Fig. 3A, panel d) as reported previously by other groups [39], [40]. Fig. 3B provides a semi-quantitative representation of the results in Fig. 3A by counting the relative distribution of the Rev-mCherry fusion proteins in the nucleus vs. cytoplasm of 100 transfected cells. These experiments were done in the presence of 5 µg/ml actinomycin D, which reduces the strength of the endogenous Rev NLS and therefore allows for the detection of relatively weak NESs in this reporter construct [39], [41], [42].

Our results so far show that NiV-M harbors a putative bi-partite NLS and two leucine/isoleucine-rich stretches that are important for nuclear export as suggested by mutagenesis studies. However, only the first leucine/isoleucine rich motif acts as a *bona fide* nuclear export signal in the context of a heterologous protein. These nuclear import/export phenotypes were recapitulated when we examined the localization of GFP-fused Mwt and NLS/NES mutants (Fig. S3).

### Nuclear localization of NiV-M correlates with budding

**Figure 4:**
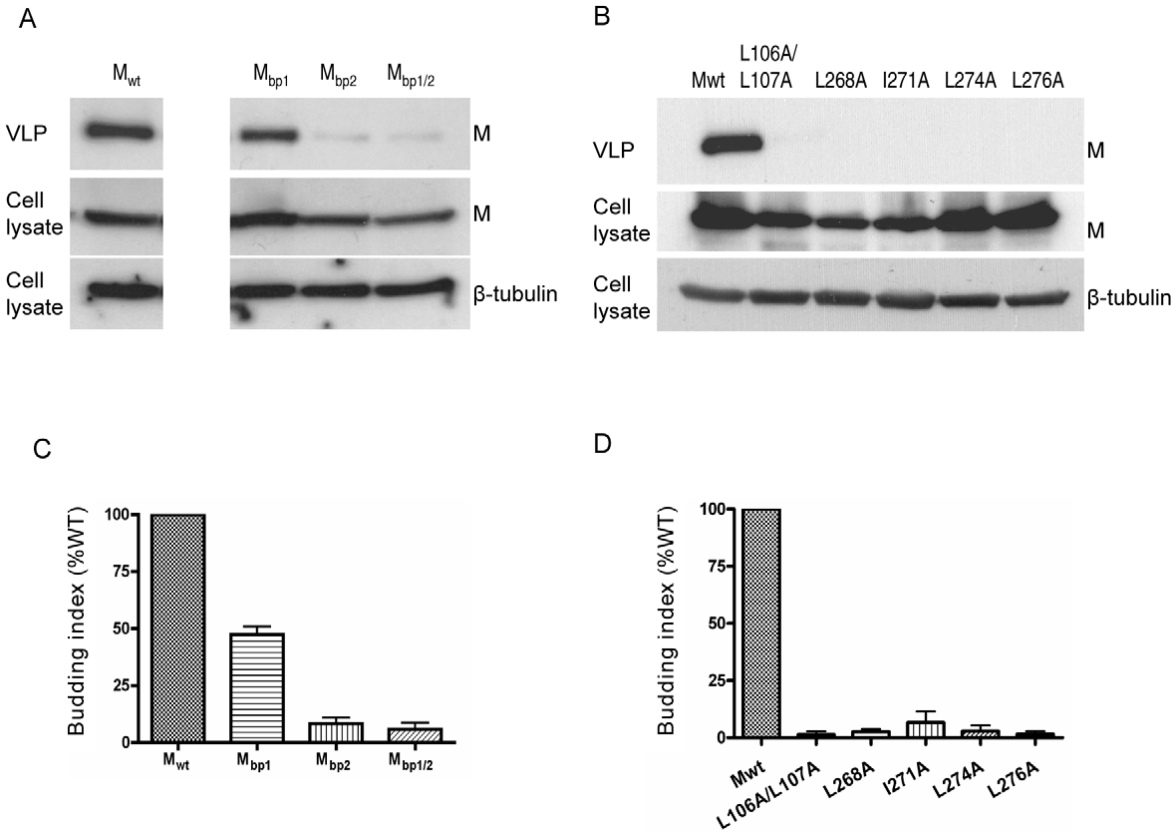
Correlation between the nuclear localization of NiV-M and VLP budding. Viral-like particles were harvested from culture supernatants of cells expressing wild-type NiV-M, NLS mutants (**A**) or NES mutants (**B**) at 24 hpt as described in *Materials and Methods*. VLPs and the corresponding cell lysates were immunoblotted with an anti-FLAG antibody. The cell lysate blots were then stripped and re-probed with an anti-β-tubulin antibody as loading control. Representative results are shown in (A) and (B). (**C**) and (**D**) show the quantification of the budding index for the indicated wild-type and mutant NiV-M proteins as described in *Materials and Methods*. Error bars were calculated from three independent experiments. M mutants that were deficient in either nuclear import or export were also deficient in budding.

The most important known function of viral matrix proteins is to mediate viral assembly and budding [7], [9]. Indeed, NiV-M, when expressed by itself in the cell, is able to form viral-like particles (VLPs) that spontaneously bud into the supernatant [10], [11], [12]. We confirmed that both 3XFLAG-tagged M and GFP-M were functional in a VLP budding assay (Figs. S4 and S5), although 3XFLAG-tagged M seemed to bud at reduced levels compared to the untagged M, especially at lower concentrations of transfected DNA. However, at concentrations we normally use for the VLP budding assay (1–2 µg of DNA), the budding index of 3XFLAG-M was not dramatically lower than untagged M. Since NiV-M was first localized to the nucleus before re-localizing to patches on the plasma membrane (Fig. 1), we sought to determine whether the nuclear-cytoplasmic trafficking of M is important for its ability to bud. We first examined the VLP budding of the NLS mutants (Fig. 4A) and found that, interestingly, the nuclear localization of M correlates with its ability to bud. M_bp1_, which had a mild nuclear exclusion phenotype, formed VLPs at a moderately reduced level compared to wild-type M, whereas M_bp2_ and M_bp1/2_, which were more deficient in nuclear import, were also more severely impaired in their abilities to bud. Fig. 4B shows that all the NES mutants were also deficient in budding, presumably due to their nuclear retention and consequentially their inability to reach the plasma membrane where budding occurs. The budding phenotype of the NLS and NES mutants were quantified by determining their budding index as described in *Materials and Methods* and shown in Fig. 4C and 4D, respectively.

The budding defect exhibited by the NLS and NES mutants is likely due to their nuclear import or export phenotypes rather than the disruption of their conformational integrity, as the budding defective mutants can associate and oligomerize with wild-type M (Fig. S6) and be rescued into VLPs by wild-type M (Fig. S7). Thus far, our data suggest that nuclear-cytoplasmic trafficking contributes to the eventual ability of NiV-M to bud.

### A conserved lysine residue plays dual roles in regulating M nuclear-cytoplasmic trafficking

Functional NLSs have been described in the matrix protein of two other paramyxoviruses, namely human respiratory syncytial virus and Newcastle disease virus [18], [22]. Our finding that NiV-M possesses a putative NLS spurred us to look at the matrix proteins of other paramyxoviruses to determine the degree to which this motif might be conserved.

**Figure 5:**
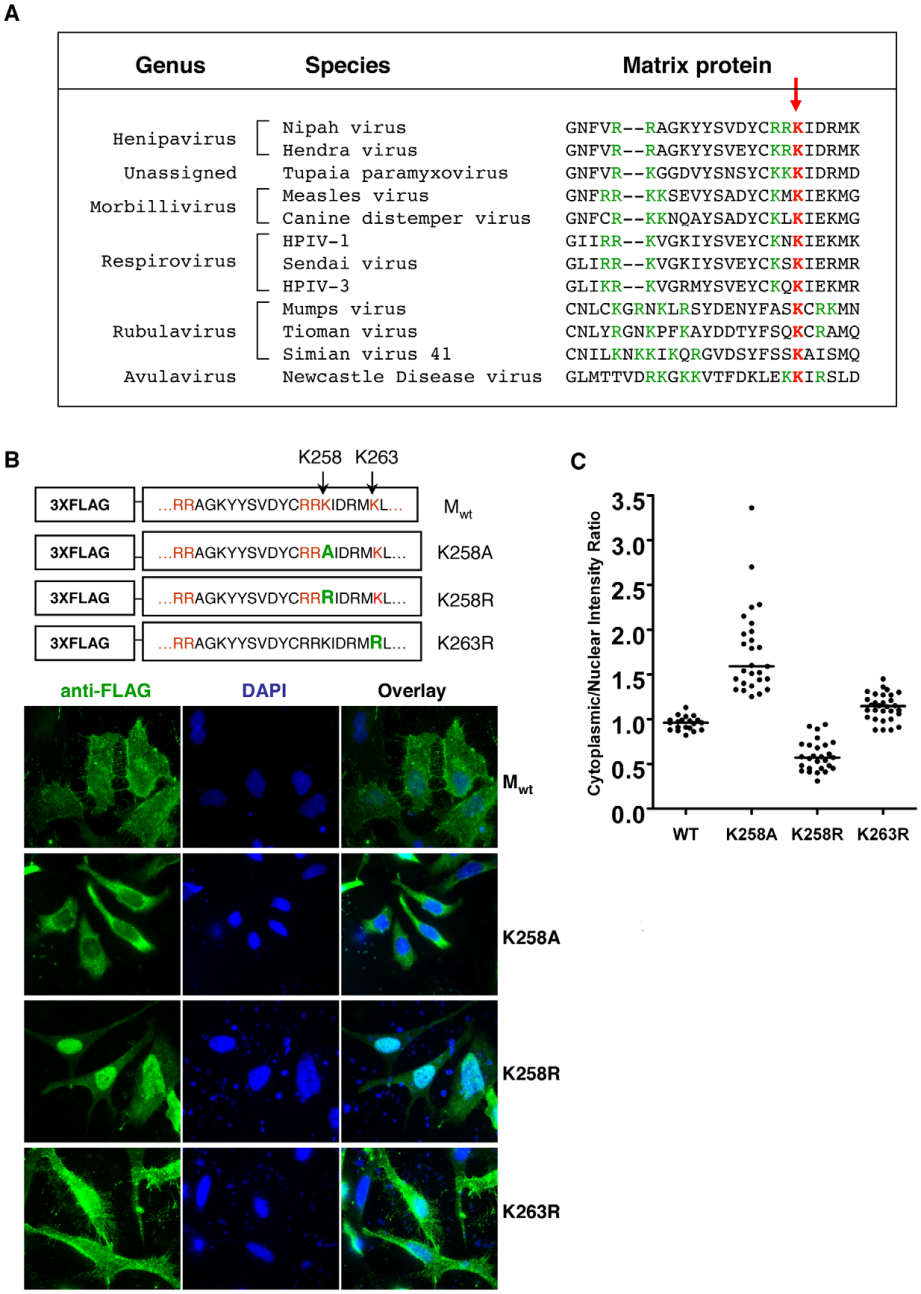
Dual functions of critical residue K258 in regulating NiV-M nuclear-cytoplasmic trafficking. (**A**) The matrix protein sequences of twelve viruses from different genera within the family *Paramyxoviridae* were aligned using CLUSTAL W (version 1.83). Positively charged amino acid residues that conform to the consensus for bipartite NLSs are colored green. The red arrow points to the lysine residue conserved among all twelve viruses. (**B**) K258 in NiV-M was mutated to alanine or arginine using site-directed mutagenesis. As control, K263, a non-conserved lysine in the vicinity of K258, was also mutated to arginine. HeLa cells transfected with the indicated constructs were stained with mouse anti-FLAG antibody and DAPI. K258A was excluded from the nucleus, whereas K258R was concentrated in the nucleus. The localization of K263R was similar to wild-type M. Quantification of cytoplasmic/nuclear fluorescence intensity ratios is shown in (**C**).

We aligned the matrix protein sequences of twelve viruses from different genera within the family *Paramyxoviridae*. Interestingly, in the same region where we identified the bipartite NLS in NiV-M, all twelve viruses had clusters of positively charged amino acids that could potentially function as bipartite NLSs (Fig. 5A). Specifically, the lysine residue in the second part of the bipartite NLS (K258 in NiV-M) was absolutely conserved, suggesting that the lysine itself, and not just the positive charge, might serve important functions. We therefore mutated K258 in NiV-M to an alanine versus an arginine.

As expected, the loss of positive charge impaired the function of the NLS. The K258A mutant was largely excluded from the nucleus, confirming that K258 was a critical part of the bipartite NLS (Fig. 5B). However, the K258R mutant, which retains the positive charge and should therefore have a localization pattern similar to wild-type M, gave a phenotype exactly the opposite of K258A. K258R was retained in the nucleus. This phenomenon was specific to K258, as mutating a nearby non-conserved lysine residue (K263) to arginine did not give an obvious nuclear retention phenotype (Fig. 5B). As in Fig. 2C, the C∶N ratios determined for each of these mutants confirmed the visual phenotypes observed (Fig. 5C). Thus, compared to Mwt, the nuclear-excluded K258A mutant had C∶N ratios significantly greater than 1 while the nuclear retained K258R mutants had C∶N ratios significantly less than 1 (p<0.0001 for both comparisons, unpaired t-test). This confirms the importance of the lysine residue itself at position 258 and suggests that potential modification(s) on K258 might be important for nuclear export. When K258 was mutated to arginine, the positive charge still allowed for nuclear import. However, arginines cannot be modified the same way that lysines are. Therefore, the K to R mutation could potentially compromise functions associated with the modified K258.

### Ubiquitination regulates NiV-M nuclear-cytoplasmic trafficking

Lysine residues could be modified in different ways including ubiquitination, SUMOylation and acetylation. Interestingly, ubiquitination has previously been shown to be involved in the nuclear-cytoplasmic trafficking of cellular proteins such as NF-kB and p53 [35]. Specifically, monoubiquitination on the C-terminus of p53 regulates its nuclear export [32], [33]. Thus, we asked if NiV-M might exploit similar pathways for its nuclear-cytoplasmic trafficking behavior.

To test whether ubiquitin was involved, we took advantage of a well-characterized proteasome inhibitor, MG132. MG132 blocks proteasome-dependent degradation of poly-ubiquitinated proteins, thus depleting the cellular pool of free ubiquitin for new conjugations. It has previously been shown to inhibit retroviral budding, presumably because ubiquitin is required for a late step during viral assembly and egress [43], [44], [45], [46].

**Figure 6:**
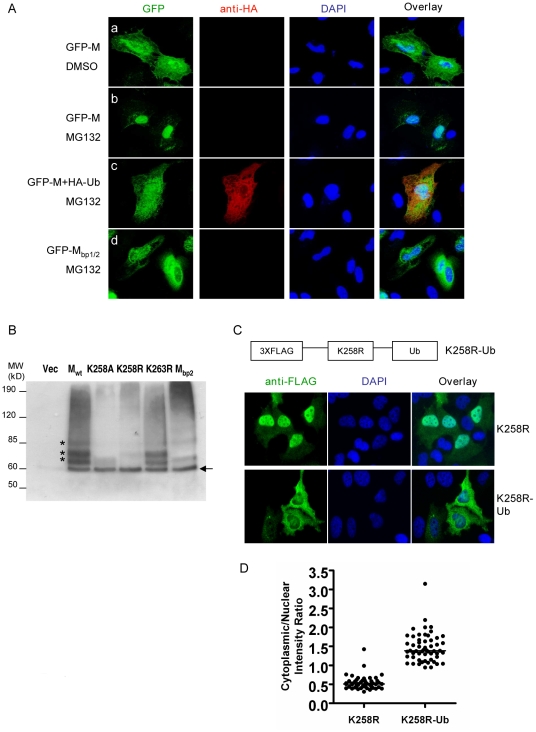
Ubiquitination regulates NiV-M nuclear export. (**A**) Ubiquitin depletion by MG132 treatment inhibits M nuclear export. HeLa cells were transfected with GFP-M alone (panels a and b), GFP-M plus HA-Ub (panel c) or GFP-M_bp1/2_ (panel d). 24 hpt, cells were treated with 50 µM MG132 or DMSO as indicated, fixed 6 hrs later with 2% paraformaldehyde, stained with DAPI as well as a mouse anti-HA antibody followed by Alexa594-conjugated goat-anti-mouse secondary antibody to identify cells expressing HA-Ub, and imaged on a fluorescent microscope. Representative images are shown. (**B**) Ubiquitination patterns of wild-type M and the indicated mutants. HEK293T cells were co-transfected with HA-Ub (in which all the lysines were mutated to arginines to specifically look at monoubiquitination) and the indicated 3XFLAG-tagged M mutants or empty vector as control. M was immunoprecipitated as described in *Materials and Methods* and the ubiquitinated species were detected by immunoblotting using an anti-HA antibody. The banding patterns of K258A, K258R and M_bp2_ were different from Mwt, whereas K263R was similar to Mwt. (**C**) Mimicking monoubiquitination restores nuclear export to K258R. One copy of ubiquitin was fused in frame to the C-terminus of 3XFLAG-K258R, and HeLa cells expressing K258R or K258R-Ub were stained with an anti-FLAG antibody. Quantification of the cytoplasmic/nuclear fluorescence intensity ratio for each mutant is shown in (**D**). There is significant difference between the localization patterns of K258R and K258R-Ub (p<0.0001, unpaired t test).

MG132 treatment resulted in the nuclear retention of GFP-M fusion protein (Fig. 6A, panel b) reminiscent of the phenotype of the K258R mutant (Fig. 5B). Similar results were obtained when another proteasome inhibitor, bortezomib, was used (Fig. S8). Overexpressing an HA-tagged ubiquitin (HA-Ub) in the cells was able to reverse the effect of MG132 (Fig. 6A, panel c), confirming that MG132's effect on the nuclear retention of NiV-M was indeed due to its effect on depleting the cellular pool of free ubiquitin, and suggesting that the ubiquitination of M is important for its nuclear export. As a specificity control, GFP-M_bp1/2_, which is impaired in nuclear import (Fig. S3), did not accumulate in the nucleus upon MG132 treatment (Fig. 6A, panel d).

To biochemically detect the ubiquitinated NiV-M, we co-transfected triple-FLAG tagged M_wt_ or the indicated mutants with HA-Ub. Since polyubiquitination is usually associated with proteasome-dependent protein degradation, whereas monoubiquitination serves regulatory functions, we wanted to specifically look at the monoubiquitinated M species by using a mutated version of ubiquitin in which all the lysine residues are changed to arginines [33]. We immunoprecipitated M with an anti-FLAG antibody and detected the ubiquitinated species with an anti-HA antibody (Fig. 6B). For M_wt_, there were at least four distinct bands at ∼8 kD intervals starting from 60 kD which represents the first monoubiquintinated band above the size of unconjugated 3XFLAG-M (Fig. 6B, arrow). These bands likely represent M monoubiquitinated on four different lysine residues. When the same experiment was performed using K258A or K258R mutant, the banding patterns were different from M_wt_. The bottom band (indicated by the arrow) was the same for M_wt_ as well as the mutants, likely indicating monoubiquitination on a lysine residue other than K258. The three bands above it, however, were significantly reduced in the mutants compared to the wild-type. These bands likely represent ubiquitinated K258 as well as other lysines whose ubiquitination depends on K258. It was not totally unexpected that the banding patterns of K258A and K258R were not exactly identical. One of them was in the cytoplasm while the other in the nucleus, where distinct ubiquitination machineries might account for the difference. As controls, K263R had the same banding pattern as wild-type, whereas M_bp2_, in which all three basic amino acid residues in the second part of the bipartite NLS including K258 were simultaneously mutated to alanines, showed a banding pattern similar to K258A.

We therefore hypothesized that ubiquitination on K258 in the nucleus might be necessary for the subsequent nuclear export of NiV-M. The altered subcellular localization of the K258R mutant was likely due to its lack of ubiquitination. To test this hypothesis, we constructed a fusion protein with one copy of ubiquitin fused in-frame to the C-terminus of K258R to mimic monoubiquitination [47], [48], [49], [50]. It has previously been shown that fusion to ubiquitin induces the nuclear export of p53 but had no effect on Max, a nuclear protein known not to be regulated by monoubiquitination [33]. Fusion to ubiquitin was able to restore nuclear export to K258R. While K258R was largely retained in the nucleus, the K258R-Ub fusion protein was clearly more cytoplasmic although in some cells, it was evenly distributed between the nucleus and cytoplasm (Fig. 6C). Fig. 6D quantifies the nuclear∶cytoplasmic ratios of K258R and K258R-Ub in ∼40–50 cells and confirms the visual phenotypes seen in Fig. 6C.

### K258 is important for the membrane association and budding of NiV-M

**Figure 7:**
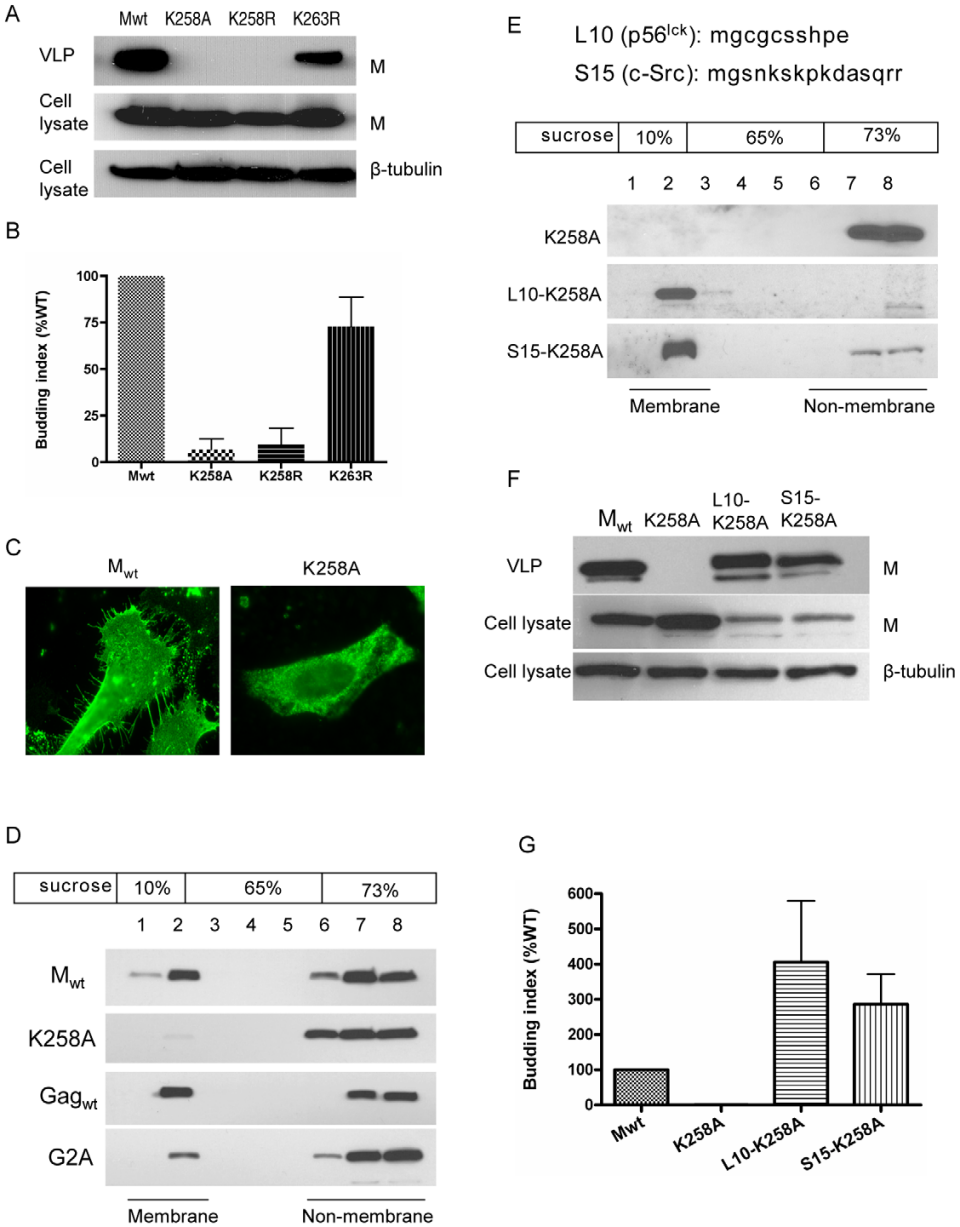
K258 is critical for NiV-M membrane association and budding. (**A**) NiV-M K258 mutants are deficient in VLP budding. VLP and cell lysate samples were prepared from cells expressing wild-type M, K258A, K258R or K263R at 24 hpt as described in *Materials and Methods*. Immunoblotting was performed using an anti-FLAG monoclonal antibody, then the cell lysate blot was stripped and re-probed with an anti-β-tubulin antibody as loading control. Both K258A and K258R were expressed in the cells at similar levels compared to wild-type M, but they were absent from the VLPs. The experiment was repeated three times and representative results are shown. (**B**) Quantification of the budding index for the wild-type and mutant NiV-M proteins shown in (A). (**C**) Wild-type M localized to membrane patches and fine filopodia extensions while the K258A mutant did not. (**D**) K258A is deficient in membrane association. HEK293T cells expressing wild-type NiV-M, K258A, wild-type HIV Gag, or a myristoylation mutant of HIV Gag (G2A) were harvested at 24 hpt. Cell homogenates were loaded at the bottom of a 10–73% discontinuous sucrose gradient and ultracentrifuged for 16 hrs at 100,000×g. Eight fractions were collected from the top, and proteins were extracted using methanol/chloroform prior to immunoblotting with anti-NiV-M (in the case of M_wt_ and K258A) or anti-myc (Gag_wt_ and G2A) antibodies. Membrane-associated proteins were collected at the interface between 10% and 65% sucrose as “fraction 2” as described previously [71]. (**E**) Fusion to L10 or S15, the membrane targeting N-terminal peptide sequence from p56^lck^ and c-Src, respectively, restores membrane association to the K258A mutant. Membrane flotation centrifugation was performed as in (D). (**F**) Rescue of K258A budding by L10 and S15. VLP and cell lysate samples were prepared from HEK293T cells expressing the indicated constructs and examined by immunoblotting using a rabbit anti-NiV-M antibody. The cell lysate blot was also probed with an anti-β-tubulin antibody as loading control. The experiment was repeated three times. Representative blots are shown in (F), and the quantification of the budding indices is shown in (**G**).

Since K258 plays key roles in regulating NiV-M trafficking, we asked whether it might also affect the ability of M to bud. We therefore purified viral-like particles (VLPs) from the culture supernatants of HEK293T cells expressing M_wt_, K258A, K258R, or K263R as control. Both K258A and K258R were deficient in budding, whereas K263R budded at similar levels compared to M_wt_ (Figs. 7A and B). K258R was defective in budding presumably because it was trapped in the nucleus and therefore not able to reach the plasma membrane where budding normally occurs, but it was less intuitively obvious why K258A, which was localized to the cytoplasm, was also budding-deficient.

A closer look at the microscopic images revealed that while M_wt_ localized to patches on the membrane as well as filopodia-like membrane extensions, K258A exhibited a more diffused cytoplasmic localization pattern indicative of a defect in membrane association (Fig. 7C). This was confirmed by a membrane flotation assay (Fig. 7D). While M_wt_ was distributed in both the membrane and non-membrane fractions, the K258A mutant was found almost exclusively in the non-membrane fractions. As controls, wild-type HIV Gag (Gag_wt_) and a myristoylation site mutant G2A were subjected to the same treatment. Gag_wt_ was in both the membrane and non-membrane fractions, but G2A, which lacks membrane association, was found mainly in the non-membrane fractions as described previously [51], [52].

To confirm that the budding defect of K258A was due to its lack of membrane association rather than a conformational defect that prevented its incorporation into the virions, we tried to rescue the budding of K258A by fusing membrane targeting signals to its N-terminus. L10 is the minimal signal required for targeting p56^lck^ to the lipid rafts, whereas S15 from c-Src targets proteins to non-raft membrane compartments [53], [54]. Both L10 and S15 were able to rescue the membrane association of K258A, as indicated by the presence of these fusion proteins in the membrane fractions as determined in our membrane flotation assay (Fig. 7E). Fusion to L10 and S15 also restored VLP budding to the K258A mutant (Figs. 7F and G). Indeed, a greater fraction of both L10-K258A and S15-K258A appeared to be in the membrane fractions which correlated with their increased budding index.

### Proteasome inhibitors block Nipah virus budding

**Figure 8:**
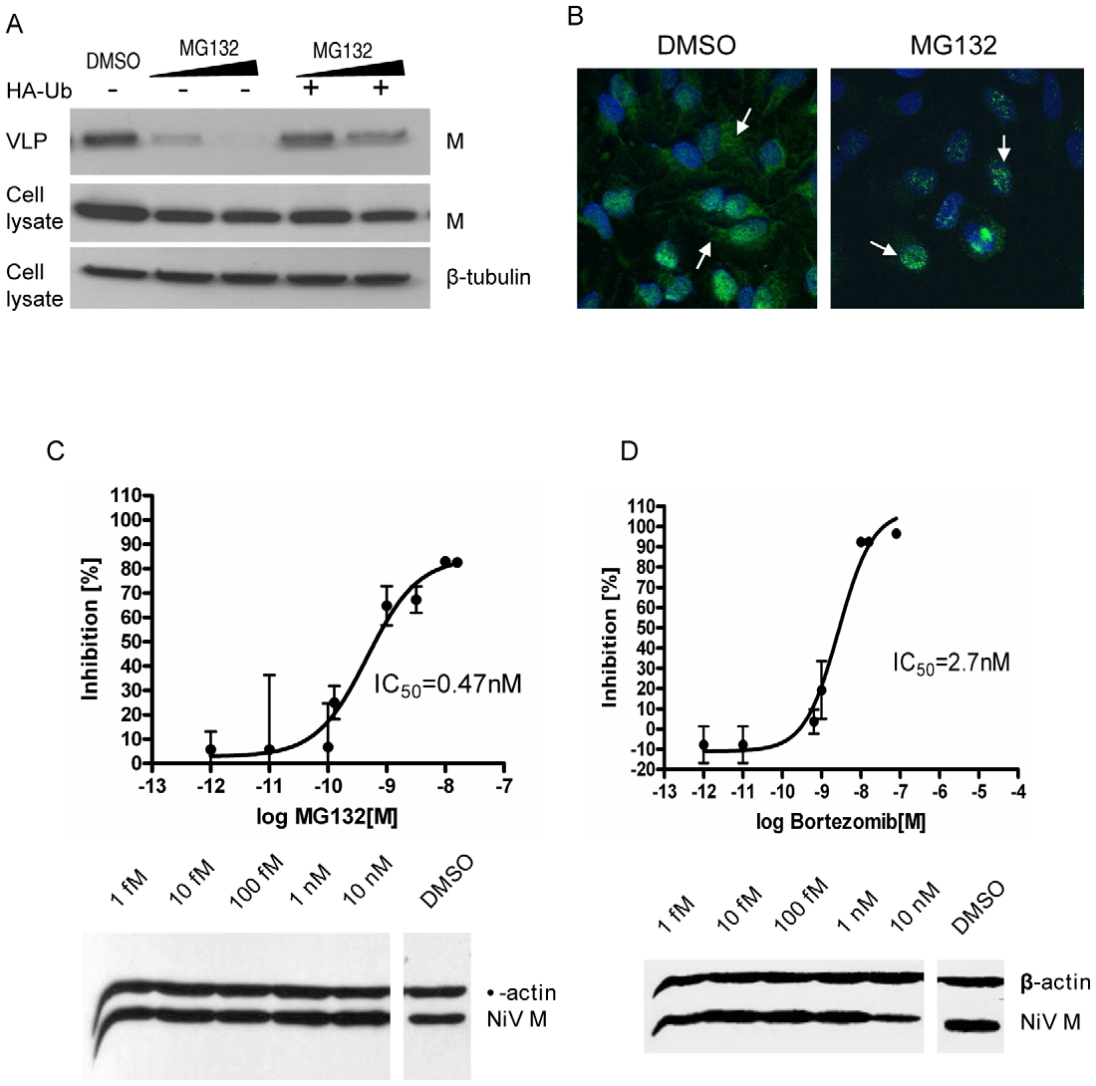
MG132 and bortezomib inhibit NiV-M nuclear export during live viral infection and reduce viral titers. (**A**) NiV-M VLP budding in the presence of MG132. HEK293T cells expressing 3XFLAG-M (left three lanes) or 3XFLAG-M plus HA-Ub (right two lanes) were incubated with DMSO, 10 µM or 50 µM MG132 for 12 hrs, and VLPs produced during this period were harvested as described in *Materials and Methods*. VLPs and cell lysates were immunoblotted with an anti-FLAG antibody, then the cell lysate blot was stripped and re-probed with an anti-β-tubulin antibody as loading control. (**B**) MG132 altered M localization during live viral infection. HeLa cells infected with Nipah virus Malaysia strain were incubated with 50 µM MG132 or DMSO for 8 hrs starting from 15 hpi. Cells were then stained with an anti-M antibody and imaged on a confocal microscope. MG132 restricted M localization to the nuclear compartment. (**C**) and (**D**) Dose-response curves of Nipah viral titers in the presence of MG132 (C) or bortezomib (D). HeLa cells were incubated with NiV for 1 hr at 37°C and then fresh growth medium. 15 hpi, serial dilutions of MG132 or bortezomib were added, yielding final concentrations ranging from 10 nM to 1 fM. Considering the short half-life of bortezomib (9–15 hrs), it was re-added 12 hrs later. Supernatants were collected at 40 hpi and viral titers were determined by plaque assay. To calculate the 50% inhibitory concentration (IC_50_), the resulting data were fit to the sigmoidal dose-response curve (GraphPad Prism software version 4.00) using the equation: % inhibition =  minimal inhibition + (maximal inhibition-minimal inhibition)/(1+10∧(LogIC_50_-Log drug concentration)). Results shown are from two independent experiments with triplicates for each data point. The infected cells were harvested, and the expression of cellular (β-actin) and viral (matrix) proteins was examined by immunoblotting.

The proteasome inhibitor MG132, which we have previously shown to inhibit NiV-M nuclear export (Fig. 6A), reduced M VLP budding in a dose-dependent manner (Fig. 8A). This inhibition was not due to potential cytotoxic effect of MG132, as both endogenous (β-tubulin) and exogenous protein (NiV-M) expression levels in the cell lysates were very similar between MG132-treated and untreated samples. The budding inhibition was also seen when a different proteasome inhibitor, bortezomib, was used (Fig. S9). Moreover, the budding inhibition by MG132 could be reversed by overexpressing HA-Ub in the cells (Fig. 8A), confirming that the inhibition was indeed due to the depletion of cellular free ubiquitin. Similar phenotypes were also observed when a different cell line, HeLa, was used instead of HEK293T (Fig. S10), suggesting that this phenomenon is not a cell-type specific effect.

Next, we wanted to confirm the effect of proteasome inhibition on M localization and activity in the context of a live viral infection. MG132 altered M localization during live Nipah virus infection (Fig. 8B), restricting M to the nucleus similar to what we have seen using the transfection system. It also reduced viral titers in a dose-dependent manner, with an IC_50_ of 0.47 nM (Fig. 8C). Under our experimental conditions, at all concentrations tested here, MG132 did not seem to be toxic to the cells as indicated by the cytotoxicity assay (Fig. S11), and the expression levels of cellular (β-actin) and viral (matrix) proteins were very similar between MG132-treated and DMSO-treated cells (Fig. 8C, lower panel). However, MG132 has limited in vivo utility due to its configurational instability [55]. Therefore, we tested another proteasome inhibitor, bortezomib, which is an FDA-approved drug for treating multiple myeloma [56]. A dose response curve for bortezomib indicates that 50% inhibition of viral infection was achieved at 2.7 nM (Fig. 8D), which is 100-fold less than the peak plasma concentration (200–300 nM) that can be reached in humans [57], [58].

## Discussion

Most negative-stranded RNA viruses, including paramyxoviruses such as Nipah virus (NiV), are known to replicate in the cytoplasm [7]. Quite unexpectedly, we found that the matrix protein of NiV transits through the nuclear compartment before reaching the plasma membrane both during live viral infection and when expressed alone in the cells.

Within the *Paramyxoviridae*, nuclear localization of matrix proteins has previously been described for Newcastle disease virus, Sendai virus and human respiratory syncytial virus [17], [20], [23]. However, to our knowledge, this phenomenon has not been directly associated with any biological functions. Our results suggest that the nuclear translocation of NiV-M is important for viral budding, as all the nuclear-excluded mutants are deficient in VLP formation (Figs. 4A, 4C, 7A and 7B). A straightforward explanation would be that post-translational modification(s) occurs in the nucleus which allows NiV-M to interact with the budding machinery once it is exported into the cytoplasm. Indeed, our data support the hypothesis that ubiquitination might be a key regulator of NiV-M intracellular trafficking and function.

The 76-amino-acid protein modifier ubiquitin is involved in the activity of many cellular as well as viral proteins [38], [59], [60]. Previous studies have shown that the ubiquitin-proteasome machinery is present in both the cytoplasmic and nuclear compartments of the cell [33], [61], [62], and a role for ubiquitin in protein nuclear/cytoplasmic trafficking has been demonstrated in the cases of cellular proteins including p53, PTEN and NF-kB [32], [33], [34], [35], [36]. We found that ubiquitination is important for NiV-M nuclear export as well as budding. Mutation of the putative ubiquitination site K258 altered M subcellular localization (Fig. 5B) and abrogated budding (Fig. 7A). These phenotypes were recapitulated when ubiquitin was depleted from the cells (Fig. 6A and Fig. 8). The involvement of ubiquitin in NiV-M budding is also reflected in our observation that overexpression of ubiquitin in the cells enhances M budding (Fig. S12). This effect is likely due to the ubiquitination of M *per se* instead of stimulating the cellular budding machinery, as the enhancement was not observed when a ubiquitination-site mutant was used.

Ubiquitin has previously been shown to be required for a late step during retroviral budding, namely the fission of virions from the cellular membrane, a process that involves the interaction between the late-domain in the Gag proteins and the cellular ESCRT complexes [37], [43], [63]. In the case of NiV-M, however, the dependence on ubiquitin seems to be via a different mechanism. Our finding that the potential ubiquitination-site mutant K258A was not membrane-associated seems to suggest a role for ubiquitin in targeting M to the plasma membrane. Plasma membrane targeting is usually mediated by N-terminal acylations such as the myristoylation of HIV Gag and the palmitoylation of Synaptosomal-associated protein of 25 kDa (SNAP-25) [64], [65]. Our sequence analysis of NiV-M did not reveal the presence of such signals, and membrane targeting is unlikely attributed solely to the interaction between M and viral transmembrane glycoproteins such as F and G, as M was able to reach the plasma membrane when expressed alone. It is possible that the ubiquitination of NiV-M might contribute to its recognition by a cellular factor that transports it to the plasma membrane. Fusing a copy of ubiquitin to the C-terminus of the K258R mutant to mimic monoubiquitination restored nuclear export (Fig. 6C), but this fusion protein was still not membrane-associated and failed to bud (data not shown), suggesting that the requirement for ubiquitin in the case of membrane targeting might be context-dependent. However, we cannot rule out the possibility that modifications other than ubiquitination might be involved.

Additionally, we found that membrane targeting positively correlates with budding. L10 and S15 peptides seem to be more potent membrane-targeting signals compared to the endogenous signal in NiV-M, as L10-K258A and S15-K258A were found predominantly in the membrane fractions whereas Mwt was present in both membrane and non-membrane fractions (Figs. 7D and F ). The more efficient membrane targeting conferred by L10 and S15 translated to higher levels of budding (Figs. 7G and H).

The cholesterol- and sphingolipid-rich membrane microdomains, or lipid rafts, have been implicated in the budding of some enveloped viruses including a few paramyxoviruses [66], [67], [68]. The fact that both L10 and S15, which target proteins to lipid raft and non-raft compartments, respectively, were equally capable of restoring NiV-M K258A budding seems to suggest that the budding of NiV-M does not require localization to the lipid rafts. This is also consistent with our observation that NiV-M localized to, but did not concentrate in lipid raft fractions (our unpublished observation). However, no conclusions can be drawn at this point as to where budding occurs during live viral infection.

NiV-M possesses two leucine/isoleucine-rich stretches, both of which are important for nuclear export, as mutating either one resulted in nuclear retention phenotypes (Figs. 2B and C). However, only one of them (amino acids 106–117) is functional in directing the nuclear export of a heterologous protein (Fig. 3). Moreover, the presence of a functional NES seems to be necessary but not sufficient for the nuclear export of NiV-M. This is demonstrated by the nuclear-retained K258R mutant, which still failed to be exported despite the presence of an intact NES. It seems that ubiquitination on K258, in addition to the NES, is required for efficient nuclear export. This is consistent with the hypothesis that ubiquitination changes the conformation of the protein, exposing the NES that is otherwise not accessible to cellular exportins [35], [49]. Alternatively, it is possible that K258 needs to be ubiquitinated for recognition by a yet-to-be-identified cellular ubiquitin-binding protein that forms an indispensable part of the nuclear export machinery. Previous studies have indicated that deleting the YMYL and YPLGVG motifs in NiV-M also results in nuclear retention [10], [12]. Since those two motifs are in proximity to the NES we identified in NiV-M, it is possible that the altered localization is due to NES masking induced by conformational changes resulting from the deletions.

Nipah virus causes fatal encephalitis in humans with high mortality rates, and there are currently no vaccines or effective therapeutics. We report here that proteasome inhibitors including MG132 and bortezomib potently reduce viral titers during live NiV infection. Bortezomib (marketed as Velcade) is an FDA-approved drug for treating multiple myeloma and mantle cell lymphoma. It is usually given to patients at a dose of 1.3 mg/m^2^ twice a week, and the mean maximum plasma concentration of the drug reaches 200–300 nM [57], [58]. Our inhibition curve (Fig. 8D) indicates that the IC_50_ of bortezomib is 2.7 nM, well below the clinically achievable plasma concentration, suggesting that it could potentially be used as an anti-viral against acute NiV infection.

Although the nuclear localization of paramyxoviral matrix proteins has been known for quite some time [17], [19], [20], [23], the biological function of this intracellular trafficking behavior remains enigmatic. Here, we provide evidence that, at least for Nipah virus matrix, nuclear transit and possible post-translational modification play critical roles in subsequent matrix-mediated viral budding. The Nipah matrix protein also illustrates the remarkably efficient use of multiple cellular trafficking machineries: that a single lysine residue in the putative bipartite NLS can serve as both a signal for nuclear import and a regulator for subsequent nuclear export. Also, the fact that the homologous lysine residue (K258 in NiV-M) in the bipartite NLS is highly conserved in all 5 genera of *Paramyxoviridae* suggests that the mechanisms described for NiV-M budding may extend to other paramyxoviruses. Finally, our findings suggest the potential use of bortezomib (Velcade) as treatment for acute henipavirus infections, or even prophylaxis in the case of high-risk exposure (such as veterinarians treating symptomatic horses in the Australian Hendra virus outbreaks). Although Velcade is an FDA-approved drug, it is not completely innocuous. However, it has the benefit of well-documented pharmacokinetic and toxicity profiles.

## Materials and Methods

### Cells and virus

VeroE6, HeLa and HEK293T cells were grown in Dulbecco's modified eagle medium (DMEM, Gibco) supplemented with 10% fetal bovine serum, 100 U/mL penicillin, 100 µg/mL streptomycin, and 1% sodium pyruvate. The Nipah virus (NiV) strain Malaysia (kindly provided by the Special Pathogens Branch, CDC, Atlanta) was propagated in VeroE6 cells. Stock virus was harvested 48 hours post infection (hpi) and virus titer was calculated using the Reed–Muench method [69].

For infection, HeLa cells were incubated with NiV for 1 hr at 37°C, and then fresh medium containing 2% FBS, 100 U/mL penicillin and 100 µg/mL streptomycin was added. For generating the dose-response inhibition curve in Figure 8, serial dilutions of MG132 or bortezomib were added at 15 hpi, yielding final concentrations ranging from 10 nM to 1fM. Considering the short half-life of bortezomib (9–15 hrs), it was re-added 12 hrs later. Supernatants were collected at 40 hpi and viral titers were determined by plaque assay.

For plaque assay, confluent monolayers of Vero cells (seeded in 12-well plates) were infected with 100 µl of serial tenfold dilutions of virus-containing cell supernatant. After 1 hr incubation at 37°C and 5% CO_2_, the inocula were removed and wells overlaid with a mixture of one part 1.0% methylcellulose and one part 2xMEM (Gibco, Invitrogen) supplemented with 2% FBS and 2% penicillin/streptomycin. The plates were incubated at 37°C and 5% CO_2_ for 3 days and then stained with 0.25% crystal violet in 10% buffered formalin. Plates were then washed and the plaques enumerated. All work with live virus was carried out under Biosafety Level 4 (BSL4) conditions in the Robert E. Shope BSL4 Laboratory, UTMB.

### Plasmids and reagents

The open reading frame encoding Nipah virus matrix protein was codon-optimized and synthesized by Geneart Inc. (Regensburg, Germany) to facilitate expression in mammalian cells. NiV-M sequence was then amplified by PCR and inserted between the HindIII and XhoI sites in the pCMV-3Tag-1 vector (Stratagene) to generate 3XFLAG-M. 3XFLAG-tagged M mutants including M_mono_, M_bp1_, M_bp2_, M_bp1/2_, L106A/L107A, L268A, I271A, L274A, L276A, K258A, K258R, R256A/R257A and K263R were generated by QuikChange site-directed mutagenesis (Stratagene). GFP sequence was fused in-frame to the N-terminus of NiV-M by overlapping PCR to generate GFP-M, and untagged NiV-M and mutants were constructed by PCR amplification and insertion into the pcDNA3.1(+) vector (Invitrogen). HA-ubiquitin constructs (wild-type and a mutant in which all the lysines were mutated to arginines) have been described previously [70] and were purchased from Addgene (Addgene plasmids 17608 and 17603). K258R-Ub was generated by fusing one copy of ubiquitin in-frame to the C-terminus of 3XFLAG-K258R. L10 and S15 sequences were fused to the N-terminus of the untagged K258A mutant by PCR using primers 5′-agaagcttgccaccatgggctgtgtctgcagctcaaaccctgaagagcccgacatcaag, 5′- ataagcttgccaccatgggtagcaacaagagcaagcccaaggatgccagccagcggcgcgagcccgacatcaag, and 5′- gtcagcctcgagtcatcagcccttcagg.

Rev-mCherry was constructed by fusing the coding sequence of mCherry at the C-terminus of HIV Rev via a GGS linker followed by a KpnI restriction enzyme site. Amino acids L78 and L81 in Rev were mutated to alanines via site-directed mutagenesis to derive RevΔNES-mCherry. RevΔNES-M^106–117^-mCherry, RevΔNES-M^264–280^-mCherry and RevΔNES-RevNES-mCherry were constructed by inserting sequences corresponding to each NES between the GGS linker and the KpnI site. All the sequences were verified by DNA sequencing. Cell transfection was performed using BioT transfection reagent per manufacturer's instructions. MG132 was purchased from Calbiochem as a 10mM stock solution in DMSO, bortezomib (Velcade) was purchased from ChemieTek, and actinomycin D was purchased from Sigma.

### Antibodies

Rabbit anti-NiV-M polyclonal antibodies were raised by immunizing rabbits with a purified peptide corresponding to amino acids 29–49 of NiV matrix protein (21st Century Biochemicals Inc.). Mouse anti-FLAG monoclonal antibody clone M2 was purchased from Stratagene. Mouse anti-HA and anti-myc (9E10) monoclonal antibodies were obtained from Covance and the Developmental Studies Hybridoma Bank at University of Iowa, respectively. Mouse anti-β-tubulin antibody was purchased from Sigma.

### Production of viral-like particles (VLPs) and quantification of the budding index

HEK293T cells were transfected with NiV-M or M mutants expression constructs. 24 hpt, culture supernatants were collected and centrifuged at 2000 rpm for 5 min to get rid of contaminating cells. The cleared supernatants were then ultracentrifuged at 30,600 rpm on an AH-650 rotor (Thermo Scientific) for 2 hrs through a 20% (w/v) sucrose cushion. VLPs pelleted at the bottom of the tubes were resuspended in lysis buffer and subjected to immunoblotting. The intensities of the bands were quantified by densitometry with a VersaDoc Imaging System (Bio-Rad), and budding index was defined as the amount of M in the VLPs divided by the amount in the cell lysate and presented as % of wt M which is set as 100%.

### Immunoprecipitation and immunoblotting

HEK293T cells expressing NiV-M or M mutants were harvested in a lysis buffer containing 50 mM Tris-HCl, 150 mM NaCl, 1% NP-40, 1 mM EDTA, 0.25% (w/w) sodium deoxycholate, 5 mM N-ethylmaleimide (NEM) and protease inhibitors cocktail (Roche). Cell lysate was clarified by centrifugation at 21,000×g for 5 min before incubation overnight with mouse anti-FLAG antibody crosslinked to protein G-conjugated agarose beads (Pierce) with 10 mg/ml dimethyl pimelimidate (DMP). Beads were then extensively washed, and the bound proteins were eluted by boiling for 10 min in SDS protein loading buffer. Proteins were separated by SDS-PAGE, transferred to PVDF membranes and analyzed using appropriate antibodies.

### Immunofluorescence microscopy and image analysis

For the live viral infection experiments, cells infected with NiV were fixed with 10% formalin for 24 hrs and removed from the BSL4. Cells were then permeabilized with 0.2% Triton X-100 in phosphate-buffered saline (PBS) for 5 min at room temperature (RT), washed with PBS and then incubated with rabbit polyclonal anti-NiV-M antibody. After extensive washing with PBS, cells were incubated with Alexa488-conjugated goat anti-rabbit secondary antibody (Invitrogen Molecular Probes) for 30 min at RT. Cells were then counterstained with DAPI. The slides were imaged on a Zeiss LSM 510 confocal microscope in the UTMB optical imaging core. For the cell transfection experiments, HeLa cells transfected with the indicated constructs were fixed with 2% paraformaldehyde, permeabilized with 0.2% Triton in PBS and stained with mouse anti-FLAG antibody followed by Alexa488 or 594-conjugated goat anti-mouse secondary antibodies. Cells were imaged on a Nikon Eclipse TE300 fluorescent microscope with MetaMorph software (Molecular Devices).

Image analysis was performed with MetaXpress software from Molecular Devices using the Enhanced Translocation module. The algorithm identifies nuclei as compartments using DAPI stain. The nuclear region was defined as the central region 20 pixels inset from the nuclear/cytoplasm boundary. The cytoplasmic region was defined as a disc beginning at the nuclear/cytoplasmic boundary and extending 5 pixels into the cytoplasm. Cells were manually included or excluded by inspection to insure that all cells included in the final scoring had the cytoplasm and nuclear regions correctly defined. A minimum cutoff intensity level was applied to ensure NiV matrix expression was sufficient. This was to exclude aberrant cell morphology and non-transfected cells. The statistic evaluated was the ratio of the average cytoplasmic region intensity to the average nuclear region intensity for each cell. Since the cytoplasmic/nuclear fluorescent intensity (C∶N) ratio for wild-type M is close to 1, C∶N ratios greater than 1 implies increased cytoplasmic retention whereas C∶N ratios less than 1 indicates increased nuclear retention. Between 10-50 cells were counted for Mwt and all mutants analyzed.

### Membrane flotation centrifugation

Membrane flotation centrifugation was performed as described previously [71]. Briefly, transfected HEK293T cells were Dounce-homogenized in cold TNE buffer containing 50 mM Tris-HCl, 150 mM NaCl, 2 mM EDTA, 0.1% 2-mercaptoethanol and protease inhibitors cocktail (Roche). Cell homogenates were clarified at 3,000 rpm for 30 min at 4°C to remove cell debris and nuclei. The cleared cell homogenate was mixed with 85% (w/v) sucrose solution to obtain 73% final concentration and loaded at the bottom of a 5 ml ultracentrifuge tube. The sample was then layered with 3 ml 65% and 0.8 ml 10% sucrose solutions and centrifuged at 100,000×g for 16 hrs at 4°C. Eight fractions (0.6 ml/fraction) were collected from the top and proteins were extracted with methanol/chloroform. Membrane-associated materials were harvested at the interface between 10% and 65% sucrose as “fraction 2”.

## Supporting Information

Figure S1 Specificity of rabbit anti-M polyclonal antibody. HeLa cells transfected with 3XFLAG-M (upper panel) or empty vector as control (lower panel) were fixed at 24 hpt and stained with rabbit anti-NiV-M antibody followed by Alexa 488-conjugated goat anti-rabbit secondary antibody. DAPI was used for visualization of the nuclei. All the pictures were acquired using the same exposure time.
(3.43 MB PDF)

Figure S2 Subcellular localization of NiV-M in transfected HeLa cells. Triple FLAG-tagged NiV-M (3XFLAG-M) was constructed by fusing three copies of the FLAG tag N-terminally to the NiV-M sequence. HeLa cells transfected with 3XFLAG-M were stained with a mouse anti-FLAG monoclonal antibody at **(A)** 12, **(B)** 16 or **(C)** 24 hrs post-transfection and imaged under 60× magnification on a fluorescent microscope. The cells were also stained with DAPI for visualization of the nuclei. At early time points, M staining was prominent in the nucleus (A), whereas at later time points, it was diffused in both the nucleus and the cytoplasm (B and C). At 24 hpt, M also localized to filamentous membrane extensions.
(3.96 MB PDF)

Figure S3 Subcellular localization of GFP-fused NiV-M and M mutants. HeLa cells were transfected with the indicated expression constructs and fixed at 24 hpt. Images were acquired under 60× magnification on a fluorescent microscope.
(1.64 MB PDF)

Figure S4 VLP budding of 3XFLAG-tagged and untagged NiV-M. HEK293T cells were transfected with the indicated amounts of DNA encoding 3XFLAG-M or untagged M. VLP and cell lysate samples were prepared at 24 hpt and immunoblotted with rabbit anti-M antibody. Arrows point to 3XFLAG-M while arrowheads indicate untagged M.
(0.24 MB PDF)

Figure S5 VLP budding of GFP-fused NiV-M. HEK293T cells were transfected with M or GFP-M expression construct. VLP and cell lysate samples were prepared at 24 hpt and immunoblotted with rabbit anti-M antibody.
(0.08 MB PDF)

Figure S6 Association between Mwt and various M mutants. HEK293T cells were co-transfected with untagged Mwt and 3XFLAG-tagged Mwt or mutants as indicated. Cells were harvested at 24 hpt, and cell lysates were subjected to immunoprecipitation using anti-FLAG monoclonal antibody M2-conjugated agarose beads (Sigma) per manufacturer's instructions. 3XFLAG peptide was used for elution, and IP samples were immunoblotted with a rabbit anti-M antibody. Arrows indicate 3XFLAG-tagged Mwt or mutants, and the arrowhead points to untagged Mwt. All the mutants tested were able to co-immunoprecipitate with Mwt.
(0.10 MB PDF)

Figure S7 Budding rescue of M mutants by wild-type M. HEK293T cells were transfected with 3XFLAG-tagged M mutants alone or together with untagged wild-type M as indicated. VLP and cell lysate samples were prepared 24 hpt. VLPs were immunoblotted with an anti-FLAG antibody to detect only the budding of the mutants, and cell lysates were probed with an anti-M antibody to visualize the expression of both untagged Mwt (arrowheads) and FLAG-tagged mutants (arrows). Mwt was able to rescue the VLP budding of all the mutants tested.
(0.10 MB PDF)

Figure S8 Bortezomib inhibits the nuclear export of M. HeLa cells expressing GFP-M were treated with the indicated concentrations of bortezomib for 6 hrs. Cells were then fixed and visualized under 60× magnification on a fluorescent microscope.
(1.14 MB PDF)

Figure S9 Budding inhibition of NiV-M by proteasome inhibitors. HEK293T cells expressing 3XFLAG-M were treated with MG132 (10 µM or 50 µM) or bortezomib (1 µM or 10 µM) for 12 hrs. VLP and cell lysate samples were immunoblotted with an anti-FLAG antibody **(A)**, and the budding indices were calculated and normalized to the DMSO control **(B)**.
(0.11 MB PDF)

Figure S10 Overexpression of ubiquitin restores budding in the presence of MG132. HeLa cells expressing 3XFLAG-M (left three lanes) or 3XFLAG-M plus HA-Ub (right two lanes) were incubated with DMSO, 10 µM or 50 µM MG132 for 12 hrs, and VLPs produced during this period were harvested as described in *Materials and Methods*. VLPs and cell lysates were immunoblotted with an anti-FLAG antibody, then the cell lysate blot was stripped and re-probed with an anti-β-tubulin antibody as loading control.
(0.12 MB PDF)

Figure S11 MG132 and bortezomib are not grossly toxic to the cells under our experimental conditions. HeLa cells were treated with MG132 or bortezomib at the indicated concentrations for 24 hrs. Culture supernatants were collected and the release of adenylate kinase was measured using a ToxiLight BioAssay kit (Lonza) per manufacturer's instructions. Results are shown as percent toxicity with DMSO background subtracted and complete cell lysis by detergent set as 100%. ND = Not Detectable.
(0.23 MB PDF)

Figure S12 Ubiquitin promotes the budding of NiV-Mwt, but not the K258A mutant. HEK293T cells were cotransfected with 3XFLAG-M or 3XFLAG-M K258A mutant plus increasing amounts of HA-Ub as indicated. 24hpt, VLPs and cell lysates were prepared as described in *Materials and Methods* and immunoblotted with an anti-FLAG antibody **(A)**. Densitometry was performed to determine the budding index **(B)** as described in *Materials and Methods*.
(0.03 MB PDF)
