# Supplementary figures and images for: Ubiquitin-Regulated Nuclear-Cytoplasmic Trafficking of the Nipah Virus Matrix Protein Is Important for Viral Budding
Source: PLoS Pathog. 2010 Nov 11;6(11):e1001186. doi: 10.1371/journal.ppat.1001186 (PMC2978725; doi:10.1371/journal.ppat.1001186)

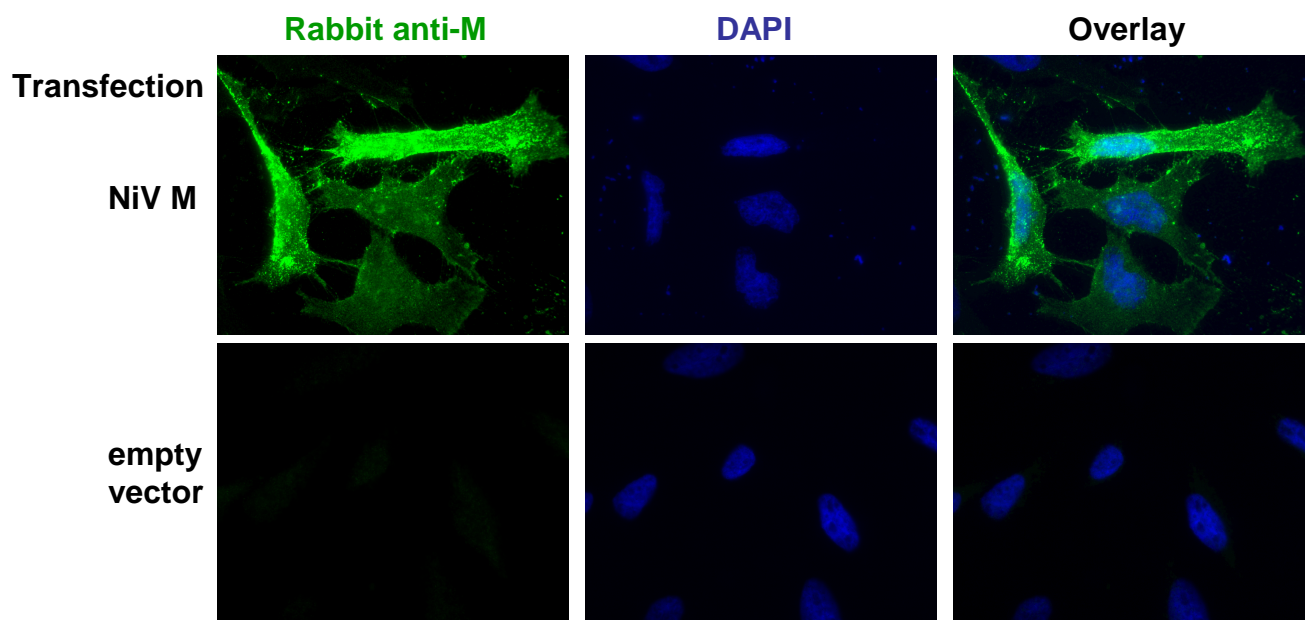

Figure S1

Supplement: Figure S1 — Specificity of rabbit anti-M polyclonal antibody. HeLa cells transfected with 3XFLAG-M (upper panel) or empty vector as control (lower panel) were fixed at 24 hpt and stained with rabbit anti-NiV-M antibody followed by Alexa 488-conjugated goat anti-rabbit secondary antibody. DAPI was used for visualization of the nuclei. All the pictures were acquired using the same exposure time. (3.43 MB PDF) [file ppat.1001186.s001.pdf]

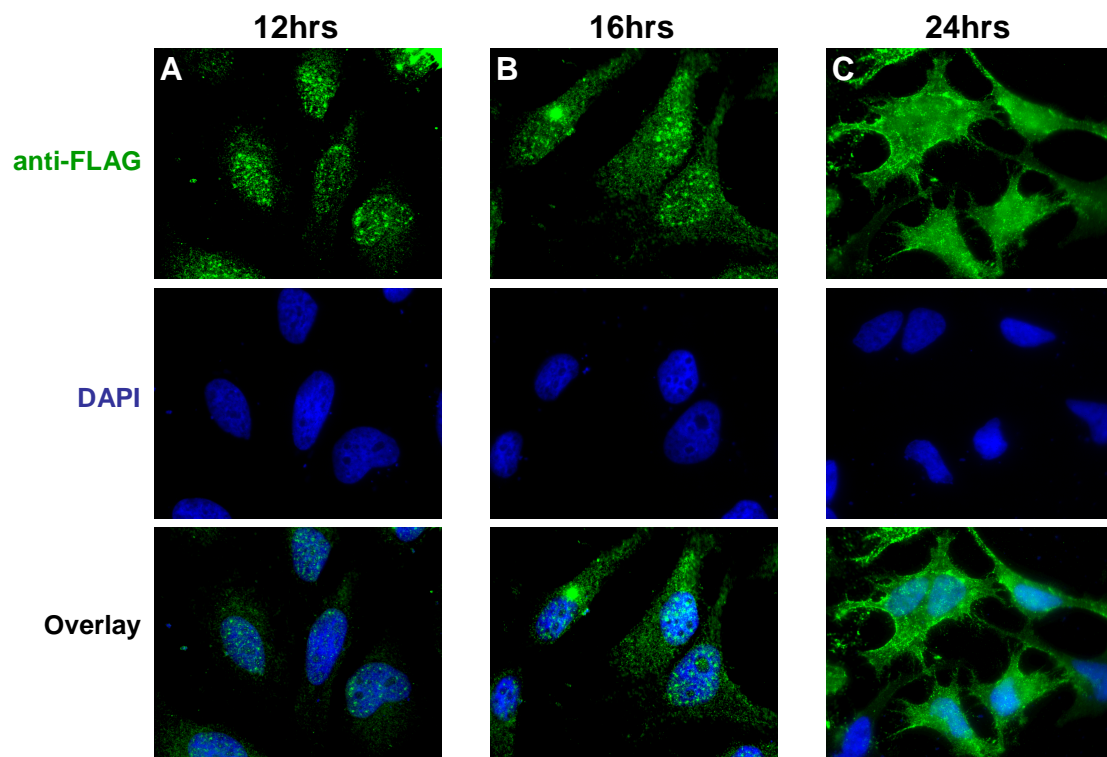

Figure S2

Supplement: Figure S2 — Subcellular localization of NiV-M in transfected HeLa cells. Triple FLAG-tagged NiV-M (3XFLAG-M) was constructed by fusing three copies of the FLAG tag N-terminally to the NiV-M sequence. HeLa cells transfected with 3XFLAG-M were stained with a mouse anti-FLAG monoclonal antibody at (A) 12, (B) 16 or (C) 24 hrs post-transfection and imaged under 60× magnification on a fluorescent microscope. The cells were also stained with DAPI for visualization of the nuclei. At early time points, M staining was prominent in the nucleus (A), whereas at later time points, it was diffused in both the nucleus and the cytoplasm (B and C). At 24 hpt, M also localized to filamentous membrane extensions. (3.96 MB PDF) [file ppat.1001186.s002.pdf]

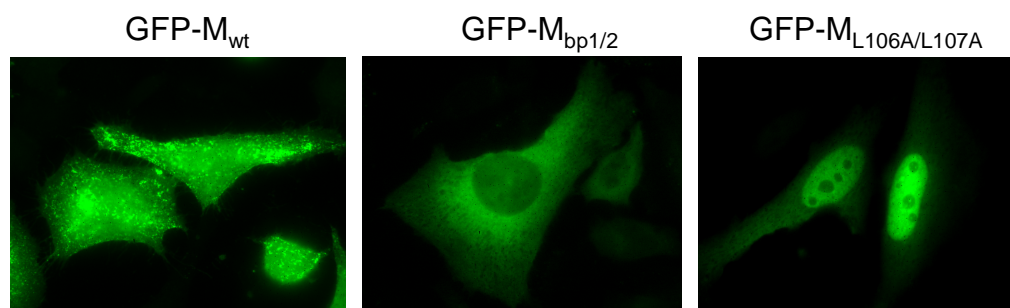

**Figure S3**

Supplement: Figure S3 — Subcellular localization of GFP-fused NiV-M and M mutants. HeLa cells were transfected with the indicated expression constructs and fixed at 24 hpt. Images were acquired under 60× magnification on a fluorescent microscope. (1.64 MB PDF) [file ppat.1001186.s003.pdf]

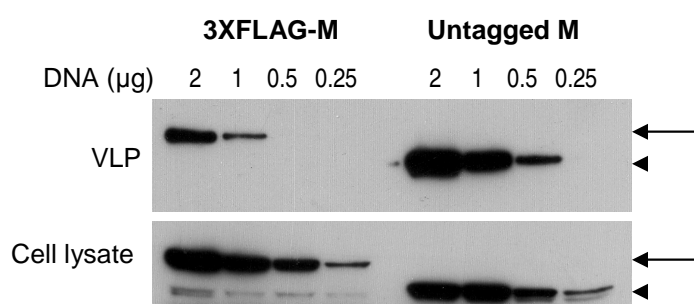

**Figure S4**

Supplement: Figure S4 — VLP budding of 3XFLAG-tagged and untagged NiV-M. HEK293T cells were transfected with the indicated amounts of DNA encoding 3XFLAG-M or untagged M. VLP and cell lysate samples were prepared at 24 hpt and immunoblotted with rabbit anti-M antibody. Arrows point to 3XFLAG-M while arrowheads indicate untagged M. (0.24 MB PDF) [file ppat.1001186.s004.pdf]

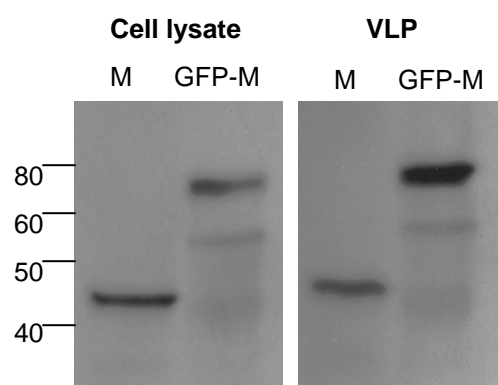

**Figure S5**

Supplement: Figure S5 — VLP budding of GFP-fused NiV-M. HEK293T cells were transfected with M or GFP-M expression construct. VLP and cell lysate samples were prepared at 24 hpt and immunoblotted with rabbit anti-M antibody. (0.08 MB PDF) [file ppat.1001186.s005.pdf]

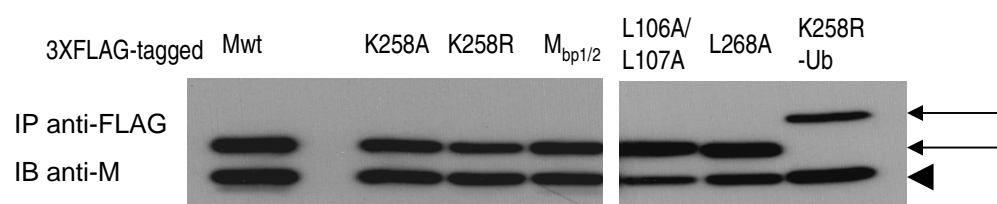

**Figure S6**

Supplement: Figure S6 — Association between Mwt and various M mutants. HEK293T cells were co-transfected with untagged Mwt and 3XFLAG-tagged Mwt or mutants as indicated. Cells were harvested at 24 hpt, and cell lysates were subjected to immunoprecipitation using anti-FLAG monoclonal antibody M2-conjugated agarose beads (Sigma) per manufacturer's instructions. 3XFLAG peptide was used for elution, and IP samples were immunoblotted with a rabbit anti-M antibody. Arrows indicate 3XFLAG-tagged Mwt or mutants, and the arrowhead points to untagged Mwt. All the mutants tested were able to co-immunoprecipitate with Mwt. (0.10 MB PDF) [file ppat.1001186.s006.pdf]

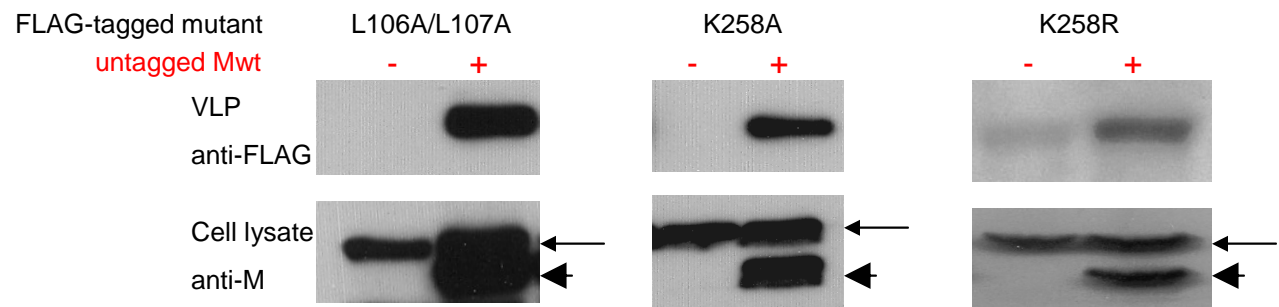

**Figure S7**

Supplement: Figure S7 — Budding rescue of M mutants by wild-type M. HEK293T cells were transfected with 3XFLAG-tagged M mutants alone or together with untagged wild-type M as indicated. VLP and cell lysate samples were prepared 24 hpt. VLPs were immunoblotted with an anti-FLAG antibody to detect only the budding of the mutants, and cell lysates were probed with an anti-M antibody to visualize the expression of both untagged Mwt (arrowheads) and FLAG-tagged mutants (arrows). Mwt was able to rescue the VLP budding of all the mutants tested. (0.10 MB PDF) [file ppat.1001186.s007.pdf]

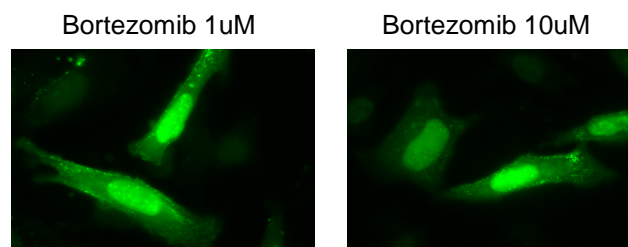

**Figure S8**

Supplement: Figure S8 — Bortezomib inhibits the nuclear export of M. HeLa cells expressing GFP-M were treated with the indicated concentrations of bortezomib for 6 hrs. Cells were then fixed and visualized under 60× magnification on a fluorescent microscope. (1.14 MB PDF) [file ppat.1001186.s008.pdf]

**A**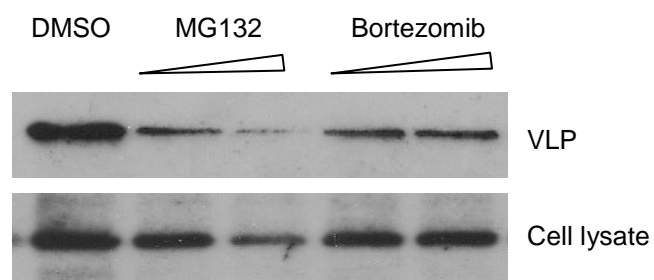**B**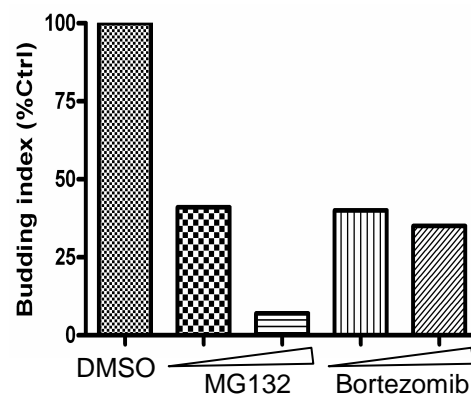**Figure S9**

Supplement: Figure S9 — Budding inhibition of NiV-M by proteasome inhibitors. HEK293T cells expressing 3XFLAG-M were treated with MG132 (10 µM or 50 µM) or bortezomib (1 µM or 10 µM) for 12 hrs. VLP and cell lysate samples were immunoblotted with an anti-FLAG antibody (A), and the budding indices were calculated and normalized to the DMSO control (B). (0.11 MB PDF) [file ppat.1001186.s009.pdf]

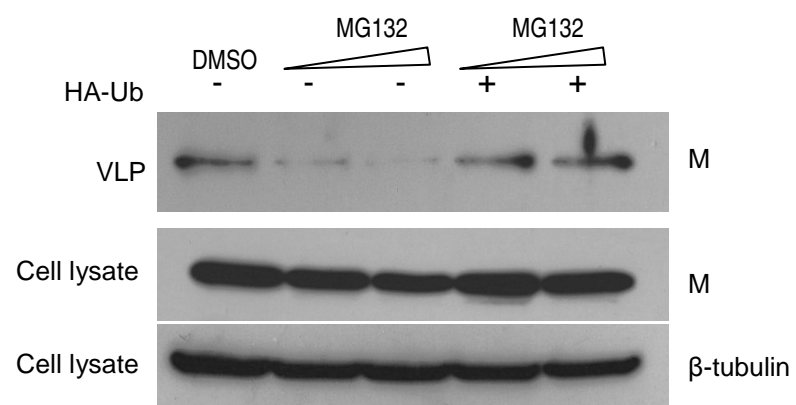

**Figure S10**

Supplement: Figure S10 — Overexpression of ubiquitin restores budding in the presence of MG132. HeLa cells expressing 3XFLAG-M (left three lanes) or 3XFLAG-M plus HA-Ub (right two lanes) were incubated with DMSO, 10 µM or 50 µM MG132 for 12 hrs, and VLPs produced during this period were harvested as described in Materials and Methods . VLPs and cell lysates were immunoblotted with an anti-FLAG antibody, then the cell lysate blot was stripped and re-probed with an anti-β-tubulin antibody as loading control. (0.12 MB PDF) [file ppat.1001186.s010.pdf]

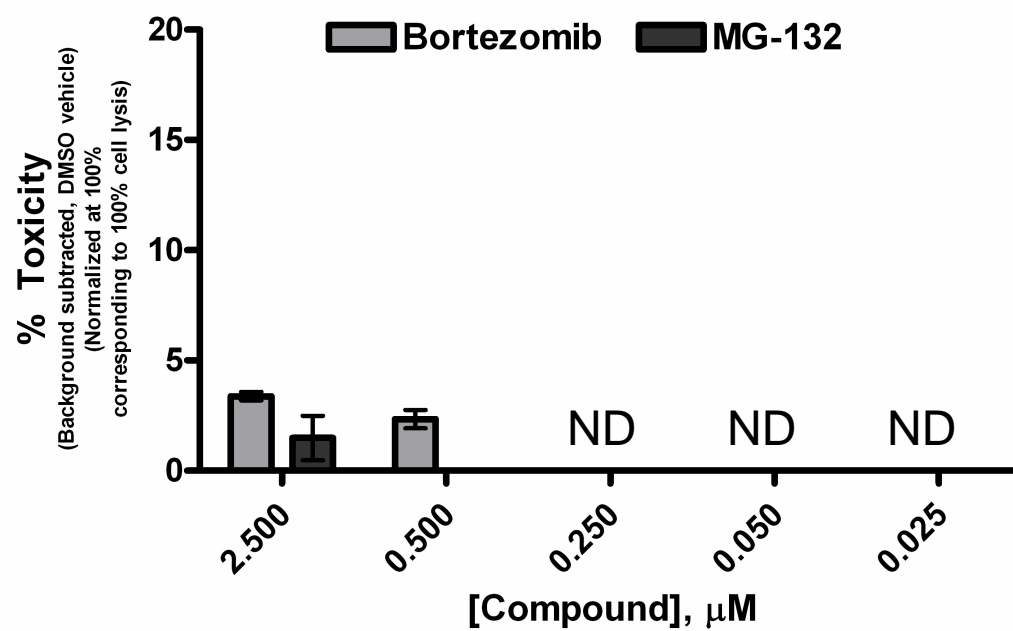

Figure S11

Supplement: Figure S11 — MG132 and bortezomib are not grossly toxic to the cells under our experimental conditions. HeLa cells were treated with MG132 or bortezomib at the indicated concentrations for 24 hrs. Culture supernatants were collected and the release of adenylate kinase was measured using a ToxiLight BioAssay kit (Lonza) per manufacturer's instructions. Results are shown as percent toxicity with DMSO background subtracted and complete cell lysis by detergent set as 100%. ND = Not Detectable. (0.23 MB PDF) [file ppat.1001186.s011.pdf]

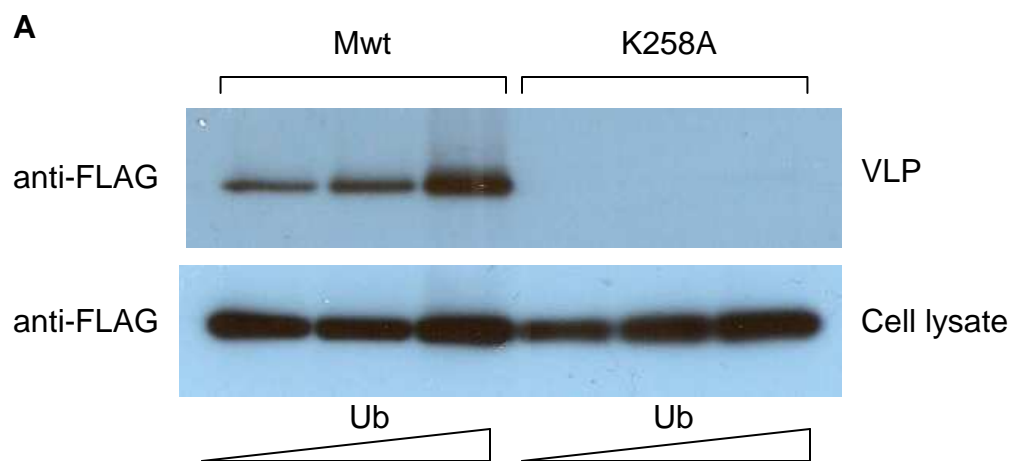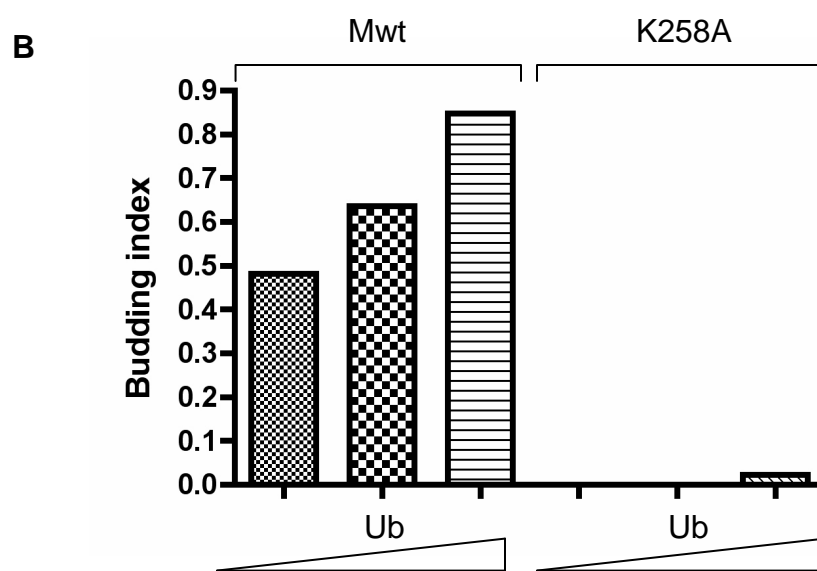

Figure S12

Supplement: Figure S12 — Ubiquitin promotes the budding of NiV-Mwt, but not the K258A mutant. HEK293T cells were cotransfected with 3XFLAG-M or 3XFLAG-M K258A mutant plus increasing amounts of HA-Ub as indicated. 24hpt, VLPs and cell lysates were prepared as described in Materials and Methods and immunoblotted with an anti-FLAG antibody (A). Densitometry was performed to determine the budding index (B) as described in Materials and Methods . (0.03 MB PDF) [file ppat.1001186.s012.pdf]
